# Microbe capture by splenic macrophages triggers sepsis via T cell-death-dependent neutrophil lifespan shortening

**DOI:** 10.1038/s41467-022-32320-1

**Published:** 2022-08-09

**Authors:** Marianna Ioannou, Dennis Hoving, Iker Valle Aramburu, Mia I. Temkin, Nathalia M. De Vasconcelos, Theodora-Dorita Tsourouktsoglou, Qian Wang, Stefan Boeing, Robert Goldstone, Spyros Vernardis, Vadim Demichev, Markus Ralser, Sascha David, Klaus Stahl, Christian Bode, Venizelos Papayannopoulos

**Affiliations:** 1 The Francis Crick Institute, Antimicrobial Defence Laboratory, London, UK; 2 The Francis Crick Institute, Bioinformatics and Biostatistics, London, UK; 3 The Francis Crick Institute, Advanced Sequencing, London, UK; 4 The Francis Crick Institute, Molecular Biology of Metabolism Laboratory, London, UK; 5 Charité Universitätsmedizin, Department of Biochemistry, 10117 Berlin, Germany; 6 Institute for Intensive Care Medicine, University Hospital Zurich, Zurich, Switzerland; 7 Department of Gastroenterology, Hepatology and Endocrinology, Medical School Hannover, Hannover, Germany; 8 Department of Anaesthesiology and Critical Care, University Hospital Bonn, Bonn, Germany

**Keywords:** Infection, Cytokines, Neutrophils, Sepsis

## Abstract

The mechanisms linking systemic infection to hyperinflammation and immune dysfunction in sepsis are poorly understood. Extracellular histones promote sepsis pathology, but their source and mechanism of action remain unclear. Here, we show that by controlling fungi and bacteria captured by splenic macrophages, neutrophil-derived myeloperoxidase attenuates sepsis by suppressing histone release. In systemic candidiasis, microbial capture via the phagocytic receptor SIGNR1 neutralizes myeloperoxidase by facilitating marginal zone infiltration and T cell death-dependent histone release. Histones and hyphae induce cytokines in adjacent CD169 macrophages including G-CSF that selectively depletes mature Ly6G^high^ neutrophils by shortening their lifespan in favour of immature Ly6G^low^ neutrophils with a defective oxidative burst. In sepsis patient plasma, these mediators shorten mature neutrophil lifespan and correlate with neutrophil mortality markers. Consequently, high G-CSF levels and neutrophil lifespan shortening activity are associated with sepsis patient mortality. Hence, by exploiting phagocytic receptors, pathogens degrade innate and adaptive immunity through the detrimental impact of downstream effectors on neutrophil lifespan.

## Abstract (web summary)

Hyperinflammation and immune dysfunction are key drivers of immunopathology in sepsis. Here the authors show microbial exploitation of phagocytic receptors is linked to triggering of sepsis and the immune cell mediated reduction in neutrophil life span.

## Introduction

Severe sepsis is triggered by systemic infection with micro-organisms that results in aberrant immune responses. It is estimated that in the year 2017 there were 48.9 million sepsis cases, of which 11 million died worldwide, accounting for ~20% of all global deaths^1^. Although fungal-induced sepsis accounts for 5% of microbial sepsis, it is the most lethal form with mortality rates exceeding 45% and overall fungal infections cause an estimated 1.6 million deaths worldwide^2,3^. The majority of these infections are caused by invasive candidiasis by *Candida albicans*, but the emergence of other pathogenic *Candida* species that are resistant to azoles is of particular concern^4^.

Septic shock is characterized by hyper-inflammation, hypotension, coagulopathy and vascular damage that drive organ failure^5^. How and where systemic cytokines are induced during sepsis is not well defined. Several damage-associated molecular pattern (DAMP) molecules such as cell-free chromatin, high mobility group protein 1 (HMGB1) and S100 proteins have also been implicated in hyper-inflammation and sepsis pathology^6–8^. Extracellular histones are cytotoxic and pro-inflammatory^6,7,9,10^, but their cellular origin and mechanisms regulating their release are unknown.

Immune dysfunction is also prominent during sepsis and is characterised by loss of cytokine responses, T cell deficiency, delayed neutrophil apoptosis and the prominence of immature neutrophils^11–14^. Neutrophils from patients with septic shock display defective antimicrobial function exemplified by lower oxidative burst capacity^14–16^. These extreme alterations in neutrophil populations persist well after septic shock and the ensuing immune deficiency negatively impacts survival following an episode of septic shock. Moreover, sepsis patients carry low T cell numbers in their spleens and prominent signs of T cell apoptosis that has been linked to Fas ligand or PD-1/PD-L1 signalling or super-antigen-mediated exhaustion^17–21^. Similarly delayed apoptosis occurs in mature neutrophils via the triggering receptor expressed on myeloid cells L4 (TREML4) receptor but the signals that regulate the specific loss of mature cells as opposed to immature neutrophils remain unknown^22^. The signals that drive neutrophil dysfunction and the links with hyperinflammation and immune dysfunction in different immune cell types are poorly understood.

In addition to causing immune deficiency, immune dysfunction may also promote pathology. For example, neutrophils are critical for controlling invading microbes but are also implicated in tissue destruction and vascular pathology during sepsis^23^ and SARS-CoV-2 infection pathology^24^. One antimicrobial protein that is specifically expressed in neutrophils is myeloperoxidase (MPO), a granule enzyme that consumes hydrogen peroxide produced by the NADPH oxidase to generate hypochlorite and other halide oxidants^25^. Patients with complete MPO-deficiencies are prone to recurrent mucosal fungal infections, but their immune deficiency is milder than chronic granulomatous disease (CGD) which is associated with mutations that disrupt the production of the upstream oxidant superoxide by the NADPH oxidase Nox2. The difference in the degree of immunodeficiency between CGD and MPO-deficient patients has suggested that MPO-derived oxidants are not as essential as the upstream oxidants. However, rare episodes of severe systemic fungal infection have also been reported in patients with complete MPO deficiency, suggesting that MPO might play more essential roles in systemic immunity^26–28^. Consistently, MPO-deficient mice are more susceptible to pulmonary infection with *C. albicans* and have increased fungal loads upon systemic challenge^29,30^. In addition to its direct microbicidal role, MPO is also required for the release of neutrophil extracellular traps (NETs). NETs control fungi but also promote vascular pathology during sepsis, suggesting that MPO could play beneficial or pathogenic roles during systemic challenge^28,30–32^.

In invasive candidiasis, fungal colonization occurs predominately in the kidneys^33^. In addition, the spleen is a key organ in the control of disseminated microbes with an array of red pulp and marginal zone (MZ) macrophages that serve to filter microbes from the circulation. The bulk of invasive candidiasis studies have focused on microbe control in the kidneys, where the spleen has recently been shown to make an important contribution. An interplay between monocytes and NK cells in the spleen is critical for the activation of neutrophils to control fungi in the kidneys^34^. Consistently, asplenic patients are at a high risk of bacterial and fungal infection^35^ and splenectomised mice are more susceptible to systemic *C. albicans* challenge^34^. MZ macrophages are comprised of an outer layer that expresses the receptors SIGN-related 1 (SIGNR1) and MARCO and the inner layer that expresses CD169. The C-type lectin receptor SIGNR1 is the murine homologue of human DC-SIGN and DC-SIGNR and is predominately expressed in the spleen and lymph nodes^36^. SIGNR1 binds *C. albicans* and augments cytokine production in macrophage cell lines^37^. Yet, it is unclear how important is fungal control in the spleen itself for survival and whether neutrophils and their oxidative armament play a central role in antifungal control in this organ.

Here, we show that MPO protects against immune dysfunction by limiting a pathogenic programme that links T cell and neutrophil dysfunction. MPO is critical for controlling pathogens in the spleen. However as the infection progresses, fungal colonization triggers T cell death that promotes neutrophil dysfunction via the release of cell-free chromatin. Our data uncover a pathway that links microbial control to immune dysfunction in the innate and adaptive immune compartments via the critical role of cell deathderived molecules.

## Results

### Myeloperoxidase regulates the onset of sepsis

**Fig. 1:**
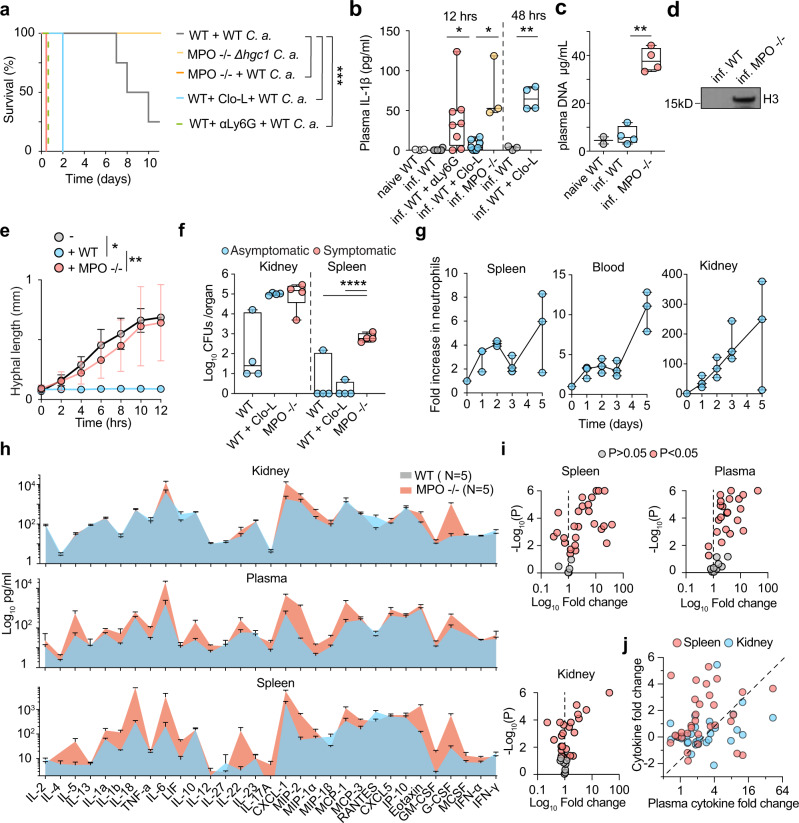
MPO control of fungal colonization in the spleen prevents the rapid onset of sepsis. **a** Survival of WT mice untreated (grey) or pre-treated with Clo-L (blue), MPO-deficient mice infected with 1 × 10^5^ WT (orange) or *Δhgc1* mutant *C. albicans* (yellow) and neutrophil-depleted WT mice (αLy6G, dotted green) (four experimental replicates with *n* = 5 mice per group). **b** Plasma IL-1β concentrations in naïve WT, infected WT or MPO-deficient, neutrophil-depleted or Clo-L-treated WT mice 12 h and 48 h post-infection (naive *n* = 3, infected *n* = 8, *n* = 3, *n* = 8 and *n* = 8 respectively). **c** Plasma DNA concentrations from naïve WT and infected WT and MPO-deficient animals, 12 h post-infection (naïve *n* = 2 and infected *n* = 4). **d** Western immunoblot of plasma histone H3 in infected WT and MPO-deficient mice, 12 h post-infection. Representative of 3 experiments. **e** Hyphal growth of WT *C. albicans* (MOI of 0.1) alone (grey) or in the presence of BM-derived neutrophils from WT (blue) or MPO-deficient mice (red) measured by time-lapse microscopy. Data represented as mean ± SD (*n* = 4 hyphae particles per condition and three individual experiments). Statistical analysis by Mann–Whitney test at 12 h. **f** Fungal load in homogenized spleens and kidneys 12 h post-infection from infected WT mice, mice pre-treated with Clo-L, or MPO-deficient (*n* = 4 mice per group). Symptomatic mice (T < 32 °C, red circles) and asymptomatic mice (blue circles). **g** Fold increase in Ly6G^+^ neutrophils in the blood, spleens and kidneys of infected WT mice, prior to infection and up to 5 days post-infection (*n* = 3 mice per group and two independent experiments). **h** Cytokine and chemokine levels in the plasma, spleen and kidney of infected WT or MPO-deficient mice 12 h post-infection, assessed by Luminex-based multiplex immunoassay. Data represented as mean ± SD of four mice per group. **i** Plots of mean fold difference in cytokine levels between MPO-deficient and WT animals and *P*-values obtained from two-sided multiple *t*-tests analysis of data in **h**. Non-significant (*P* > 0.05, grey) and significant differences (*P* < 0.05, red). Each point represents a single factor. **j** Graph depicting the ratio of each factor in MPO-deficient over WT mice in the spleen and kidneys against the respective fold change ratio in the plasma. Statistical analysis by two-sided unpaired Mann–Whitney *t*-test for single comparisons, two-tailed Log-rank (Mantel–Cox) test for survival and two-way Anova for cytokine arrays (*p < 0.05, **p < 0.01, ***p < 0.001, ****p < 0.0001).

To investigate whether MPO plays a protective or pathogenic role during systemic infection, we performed survival experiments in WT and MPO-deficient mice using a model of systemic candidiasis. WT mice infected with 1 × 10^5^ WT *C. albicans* succumbed 7–15 days post-infection, whereas MPO-deficient animals developed severe symptoms within 12 h post-infection, exhibiting a substantial decrease in body temperature (Fig. 1a, Supplementary Fig. 1a). In contrast, pulmonary challenge of MPO-deficient animals with a higher fungal load requires > 7 days to lead to serious infection^30^. The severity of the MPO-deficient phenotype was comparable to the phenotype of infected *Cybb*-deficient mice that lack the upstream NADPH oxidase activity (Supplementary Fig. 1b).

Given that low MPO expression has been reported in macrophages, we also depleted neutrophils with an anti-Ly6G antibody or macrophages with intravenous administration of clodronate liposomes (Clo-L)^38^. Neutrophil depletion resulted in acute symptoms arising within 12 h, similarly to MPO-deficient animals. By contrast, Clo-L-treated mice developed symptoms 48 h post-infection (Fig. 1a). Consistently, MPO-deficient mice exhibited elevated circulating IL-1β concentrations (Fig. 1b) and cell-free chromatin concentrations (Fig. 1c, d). IL-1β and cell-free histones were undetectable in infected WT mice at this early timepoint. The presence of circulating chromatin in MPO-deficient mice confirmed that NETs were not a major source of circulating histones.

Neutrophils are critical in controlling fungal germination. Consistently, MPO-deficient animals did not develop any symptoms after systemic challenge with the *Δhgc1* yeast-locked mutant *C. albicans* strain (Fig. 1a). Moreover, MPO-deficient bone marrow (BM) neutrophils failed to control hyphal germination and growth in vitro (Fig. 1e). By comparison, WT neutrophils suppressed hyphal growth during the first 4 h, prior to neutrophil death. Unlike human peripheral blood neutrophils, MPO-deficient murine BM neutrophils failed to release NETs under these conditions and died with condensed nuclei, indicating that the production of hypochlorous acid but not hydrogen peroxide directly suppresses hyphal growth in the absence of NETosis (Supplementary Fig. 1c). These experiments suggested that MPO-derived oxidants are critical in controlling the hyphal form to avert the rapid onset of sepsis.

To investigate which organs were afflicted by the MPO deficiency we examined the fungal load at 12 h post-infection. Systemic candidiasis in mice drives sustained fungal loads in the kidney. At 12 h post-infection, WT mice carried a low kidney fungal load, whereas MPO-deficient and Clo-L-treated mice exhibited comparably elevated fungal loads (Fig. 1 f). However, Clo-L treated animals did not exhibit symptoms of disease as opposed to MPO-deficient animals that exhibited significantly higher plasma IL-1β levels and symptoms of severe systemic inflammation such as a hunched posture, piloerection, severe hypothermia and lack of responsiveness to stimulation (Fig. 1a, b, Supplementary Fig. 1a). We noted that MPO-deficient mice carried a significant fungal load in the spleen, suggesting that the invasion of this organ could play a role in the onset of acute symptoms. Consistently, the relative numbers of neutrophils in the spleen of infected WT animals peaked rapidly in the first 48 h post-infection and the total number of cells exceeded the number of neutrophils found in the kidneys, providing further evidence that the accumulation of neutrophils in the spleen might be critical during the early stages of infection (Fig. 1g, Supplementary Fig. 1d).

To investigate the potential link between splenic colonization and hyperinflammation we analysed pro-inflammatory mediators in the plasma, spleen and kidneys of naïve and infected WT and MPO-deficient animals using luminex-based cytokine arrays. Cytokine and chemokine levels were significantly elevated and exhibited a similar pattern of induction in the spleen and blood of infected MPO-deficient mice relative to infected WT controls (Fig. 1h). In comparison to the spleen and plasma, these factors exhibited more consistent levels in the kidneys of the two groups and a different overall pattern of induction. Consistently, relative to infected WT controls, a higher number of cytokines were significantly elevated in MPO-deficient spleens and plasma than in the kidneys (Fig. 1i). Plots comparing the fold change for each cytokine in the plasma, spleen and kidney confirmed that the highest increase in cytokine levels in MPO-deficient mice occured in the spleen, followed by the plasma and with the lowest difference occuring in the kidney (Fig. 1j). Together, these findings suggested that in systemic candidiasis, neutrophils control splenic colonization and systemic inflammation in an MPO-dependent manner.

**Fig. 2:**
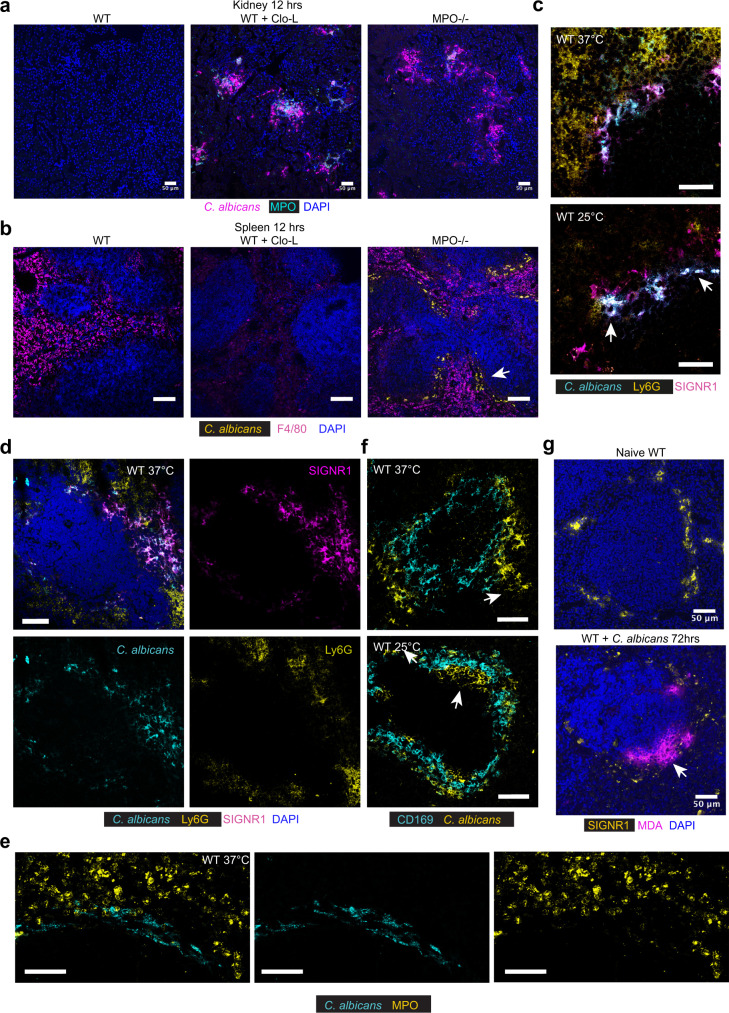
*C. albicans* are captured at the spleen marginal zone. **a**, **b** Immunofluorescence micrographs from kidneys (**a**) and spleens (**b**) of untreated WT mice or pre-treated with Clo-L, or MPO-deficient mice infected intravenously with 1 × 10^5^ WT *C. albicans*, sacrificed 12 h post-infection and stained for F4/80, *C. albicans* and DAPI. Arrow points to fungi. Scale bars: **a** 50 μm and **b** 100 μm. Data from two independent experiments and from at least four mice analysed individually. **c**–**f** Immunofluorescence micrographs from spleens of infected WT mice, 4–7 days post-infection with either a body temperature of 37 °C or 25 °C, stained for **c** *C. albicans*, Ly6G and SIGNR1, **d** *C. albicans*, Ly6G, SIGNR1 and DAPI, **e** *C. albicans* and CD169 and **f** *C. albicans* and MPO. Arrows point to fungi. Scale bars: (**c**, **d**, **e**) 50 μm, **f** 20 μm. Data from two independent experiments and four mice analysed individually. **g** Micrographs of spleens from naïve of infected WT mice, stained for DAPI, MDA, and SIGNR1. Scale bars 50 μm (*n* = 3 mice per group).

Next, we investigated the precise localization of fungi in the organs by immunofluorescence microscopy. At 12 h post infection, the kidneys of both MPO-deficient and Clo-L-treated mice were colonized in a non-selective manner, whereas in the spleens of infected MPO-deficient animals, *C. albicans* colonized predominately the marginal zone (MZ) (Fig. 2a, b). The spleen MZ is comprised of two layers of macrophages. The outer layer expresses the receptors SIGNR1 and MARCO and the inner layer expresses the receptor CD169 (Supplementary Fig. 2a). Clo-L administration had little effect on splenic macrophages within the first 24 h, but eliminated a substantial number of F4/80^+^ red pulp cells, depleted SIGNR^+^ macrophages completely and partially depleted CD169^+^ MZ macrophages, 48 h post-treatment (Supplementary Fig. 2b). WT and Clo-L-treated mice did not bear visible microbial colonies in the spleen 12 h or 24 h (not shown) post-infection which correlated with the lack of symptoms in these mice (Fig. 2a, b).

To examine how fungal invasion progresses in the spleen over the course of infection we stained samples from infected WT mice that were collected from a period ranging from 4 to 7 days post-infection. These samples originated from both asymptomatic and symptomatic mice. In asymptomatic mice with a physiological body temperature of 37 °C, fungi colocalized with SIGNR1^+^ macrophages (Fig. 2c, d). *C. albicans* and Ly6G^+^MPO^+^ neutrophils did not co-localize in the spleen (Fig. 2d, e). In symptomatic mice with a low body temperature, *C. albicans* infiltrated the inner CD169^+^ macrophage layer, suggesting that invasion within this layer correlates with the onset of pathology (Fig. 2c, f). To detect ROS production, we stained these splenic sections with antibodies against malondialdehyde (MDA) which is produced as a result of polyunsaturated fatty acid peroxidation. Prominent MDA staining was absent in naïve mice but co-localised with SIGNR1^+^ macrophages in *C. albicans* infected mice (Fig. 2g). Taken together, these data indicated that neutrophils were critical to control fungi that were captured by SIGNR1^+^ macrophages by pumping MPO-derived ROS near the MZ. The fact that MPO delayed colonization in areas that did not contain neutrophils suggested that neutrophil-derived ROS controlled hyphal growth in a non-cell-autonomous, transcellular manner.

### SIGNR1-mediated capture promotes fungal colonization and cytokine production at the spleen MZ

**Fig. 3:**
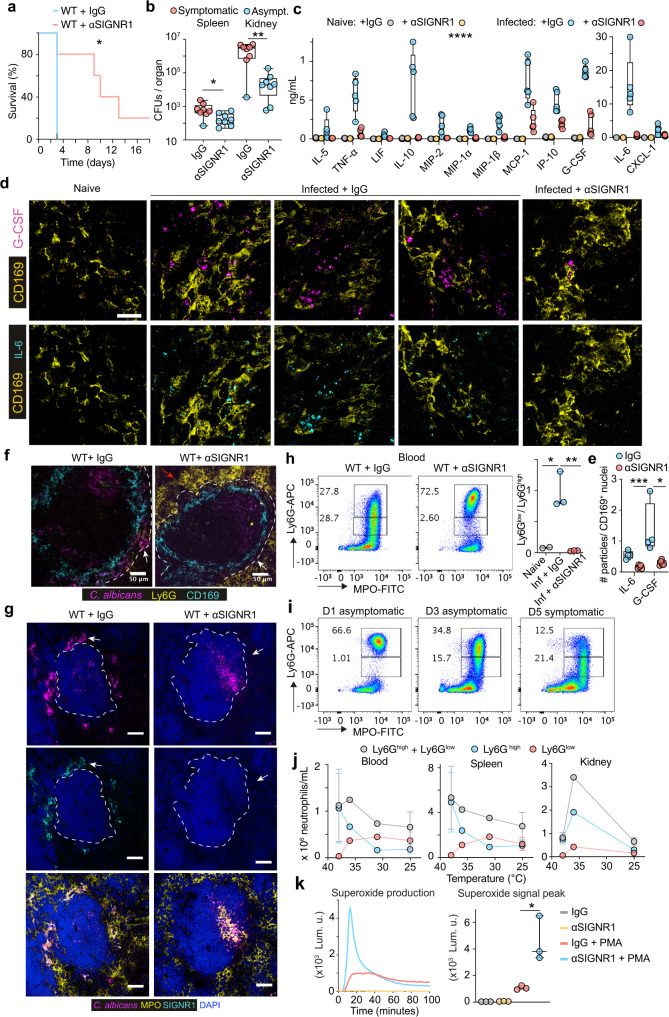
SINGR1 promotes fungal colonization, cytokine production and dysregulation of neutrophil populations. **a**–**j** WT mice pre-treated with either control antibody or an anti-SIGNR1 blocking antibody and infected intravenously with 5 × 10^5^ WT *C. albicans*: **a** Survival curves (*n* = 5 mice per group and three independent experiments). **b** Colony forming units (CFUs) 72 h post-infection with symptomatic mice (red) and asymptomatic mice (blue) (*n* = 8 mice per group). **c** Selected cytokines in plasma at 72h  post-infection detected by Luminex-based multiplex immunoassay (naïve *n* = 3 and infected *n* = 5 mice per group). **d** Representative RNA scope confocal microscopy of G-CSF and IL-6 expression in the spleens 72h  post-infection co-stained with anti-CD169 antibodies. Scale bars: 20 μm. Representative of *n* = 5 mice per group. **e** Average number of RNA scope particles / CD169 positive nuclei (DAPI) in images from 4 mice per group. Statistical analysis by unpaired Mann–Whitney *t*-test. **f**, **g** Immunofluorescence confocal micrographs of spleens from infected mice treated with control or anti-SIGNR1 antibody at 3 days post-infection, stained for **f** *C. albicans*, Ly6G, CD169, or **g** *C. albicans*, SIGNR1, MPO and DAPI. Arrows point to the MZ. Scale bars: 50 μm. Representative of *n* = 3 mice per group, and 3 independent experiments. **h** Flow cytometry of blood neutrophils at 3 days post-infection, stained for intracellular MPO and Ly6G expression. Ly6G^high^ neutrophils (top gate) and Ly6G^low^ neutrophils (bottom gate). The right panel depicts Ly6G^low^/Ly6G^high^ neutrophil ratios per animal. Gated on Live CD3^−^ CD19^−^ CD11b^+^ cells. Representative of 3 independent experiments with three mice per group. **i** Flow cytometry of blood neutrophils at 1, 3 and 5 days post-infection, stained for intracellular MPO and surface Ly6G (*n* = 3 mice per group, and 3 independent experiments). **j** Total neutrophil numbers in blood, spleen and kidneys of infected WT mice against their body temperature (*n* = 8 mice). Panel: Live/Dead blue, CD3/CD19 PerCP-Cy5.5, CD11b-PECy7, Ly6G-APC. **k** ROS production in the absence or presence of PMA by splenic neutrophils isolated 72 h post-infection. Representative superoxide production curves from one mouse per group detected by luminol bioluminescence (left) and cumulative ROS peak levels for neutrophils from three animals per group (right). Statistical analysis by unpaired two-sided Mann–Whitney *t*-test for single comparison and two-tailed Log-rank (Mantel–Cox) test for survival analysis (*p < 0.05, **p < 0.01, ***p < 0.001, ****p < 0.0001).

Next, we investigated whether the capture of fungi promotes sepsis pathology by targeting the SIGNR1 receptor. SIGNR1^+^ staining was absent in the infected kidneys indicating that the antibody would not directly impact immune responses in this organ (Supplementary Fig. 3a). SIGNR1 staining was not detectable in the spleens of mice treated with anti-SIGNR1, indicating the effective blockade of the receptor. However, the antibody did not deplete SIGNR1^+^ macrophages as they could still be detected with antibodies against MARCO (Supplementary Fig. 3b, c). For these experiments, we raised the infection dose to 5 × 10^5^ yeast particles per animal in order to facilitate the detection of a survival benefit against untreated WT mice that are more resistant to infection than MPO knockout animals. With the higher fungal dose, WT mice treated with a control IgG antibody developed severe symptoms 3 days post-infection, whereas SIGNR1 blockade delayed the onset of symptoms and extended survival by 3-fold (Fig. 3a, Supplementary Fig. 3d). SIGNR1 blockade reduced the fungal load in the spleen as well as in the kidneys, despite the absence of the receptor in this organ (Fig. 3b, Supplementary Fig. 3e). Moreover, SIGNR1 blockade reduced NET deposition in the kidneys (Supplementary Fig. 3f) and lowered the levels of most inflammatory cytokines and chemokines including IL-6 and G-CSF, in the spleen, blood and kidneys 3 days post-infection (Fig. 3c, Supplementary Fig. 4). To determine which splenocytes produced cytokines in a SIGNR1-dependent manner, we stained splenic sections for IL-6 and G-CSF mRNA transcripts by fluorescent in situ hybridization (RNAscope). Notably, both cytokines were upregulated specifically in CD169^+^ macrophages in infected IgG-treated mice and the transcript levels were reduced in anti-SIGNR1 treated mice (Fig. 3d, e). Hence, SIGNR1-mediated microbe capture promotes local and systemic cytokine production to accelerate the onset of sepsis pathology in organs that do not express the receptor.

### SIGNR1 regulates neutrophil populations

Next, we investigated the impact of SIGNR1 blockade on fungal capture in the spleen. Anti-SIGNR1 treatment prevented the sequestration of *C. albicans* in the spleen MZ but microbes could be detected in other splenic areas, such as the white pulp (Fig. 3f, g). In addition, there was a notable absence of Ly6G staining in infected control IgG-treated mice whereas robust Ly6G staining was still present in anti-SIGNR1-treated animals (Fig. 3f). The prominent MPO staining in the infected spleens of control IgG-treated mice suggested that neutrophils were still present in large numbers but lacked Ly6G expression (Fig. 3g). To further test this hypothesis, we analysed neutrophil populations in the blood, spleens and kidneys by flow cytometry, tracking CD11b, MPO and Ly6G. The administration of anti-SIGNR1 antibody did not alter the total neutrophil numbers in the blood on the day of infection nor did it affect neutrophilia 24 h post-infection (Supplementary Fig. 5a). However, at 3 days post-infection, control animals contained a large fraction of neutrophils that exhibited high MPO but low Ly6G expression (Fig. 3h, i, Supplementary Fig. 5b). In contrast, mice treated with anti-SIGNR1 antibody maintained a significantly higher Ly6G^high^ population in the blood, spleen and kidneys. The shift towards an elevated Ly6G^low^/ Ly6G^high^ neutrophil ratio in the blood, spleen and kidneys occurred gradually and preceded the onset of sepsis symptoms (Fig. 3i, Supplementary Fig. 5c, d). We also plotted the change in neutrophil populations against the corresponding body temperature in an experiment where WT mice became gradually sick and were sacrificed at an intermediate timepoint that contained a mixed group of symptomatic and asymptomatic mice. These plots showed that although the total neutrophil population decreased by ~30%, there was a near 10-fold decrease in Ly6G^high^ neutrophils and a trend for an increase in Ly6G^low^ cells as body temperature decreased (Fig. 3j).

Next, we explored whether the shift in neutrophil populations influenced the antimicrobial capacity of neutrophils. We evaluated superoxide production by the NADPH oxidase in neutrophils isolated from the spleens of infected control and anti-SIGNR1-treated mice 3 days post-infection. Neutrophils from anti-SIGNR1-treated mice produced a potent ROS burst upon stimulation with phorbol-myristate-acetate (PMA), whereas neutrophils from isotype control-treated mice exhibited a defective ROS burst (Fig. 3k). Interestingly, SIGNR1 blockade failed to improve the survival of infected MPO-deficient mice even at 100-fold lower infection doses, confirming that the beneficial impact of SIGNR1 blockade is mediated by regulating oxidative neutrophil effector function (Supplementary Fig. 5e). These data indicated that SIGNR1-mediated fungal capture promotes splenic MZ colonization and neutrophil dysfunction by interfering with ROS production in neutrophils. The systemic impact SIGNR1 exerts on neutrophil antimicrobial fitness explains why SIGNR1 blockade affected microbial clearance in organs where the receptor is not expressed, such as the kidneys.

### SIGNR1 promotes T cell death and chromatin release

**Fig. 4:**
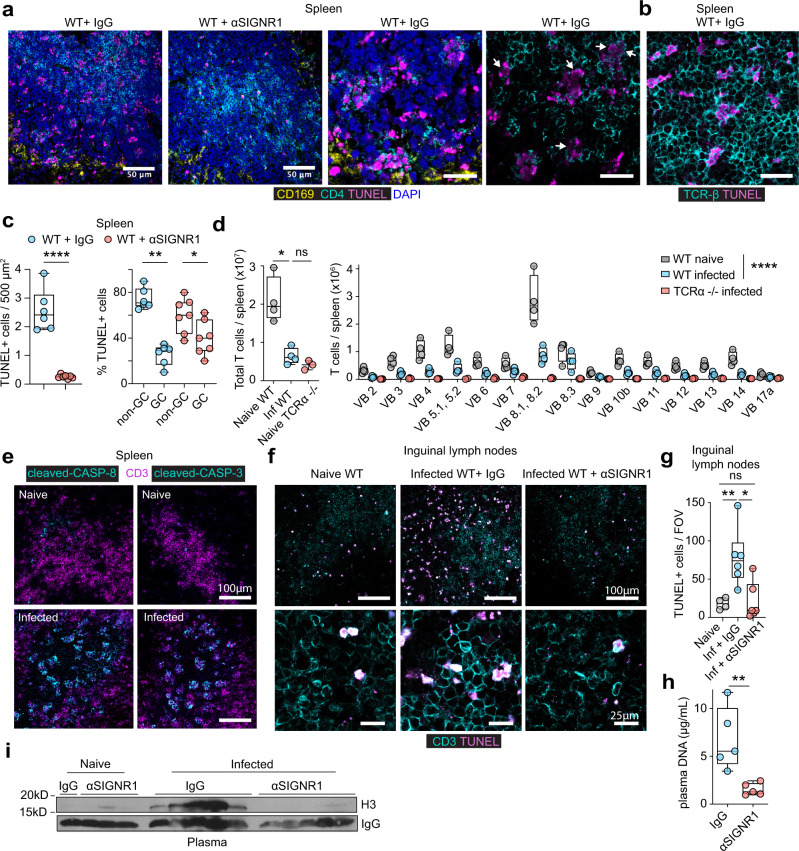
SINGR1 regulates T cell death. **a** Immunofluorescence micrographs from the spleens of WT mice infected with 5 × 10^5^ WT *C. albicans*, 72 h post-infection that were pre-treated with either control or anti-SIGNR1 antibody and stained for CD169, TUNEL, CD4 and DAPI. Representative images of three animals per group. Scale bars: 50 μm (left panels) and 20 μm (right panels). **b** Staining of spleens from symptomatic WT infected animals with TUNEL and anti-TCR-β antibodies. Representative images of three animals per group, three independent experiments. Scale bar: 20 μm. **c** Quantification of TUNEL^+^ cells in the white pulp of the spleen per 500 μm^2^ (left panel), and distribution of TUNEL^+^ positive cells within or outside germinal centers (GC) from three mice and seven fields of view. **d** Flow cytometric analysis of the numbers of total T cells (left) and TCR Vβ subset T cells (right) in the spleens of naïve WT, naive TCRα-deficient mice (negative control) and WT mice infected with 5 × 10^5^ WT *C. albicans*, 72 h post-infection. Gates were set on Live CD19^−^ CD3^+^ cells. Panel: Live/Dead blue, CD19 PerCP-Cy5.5, CD3-PE, TCR Vβ-FITC Average and SD of 3 (TCRα-deficient) and four (WT) mice are shown. **e** Immunofluorescence micrographs from spleens of naïve WT mice or infected with 5 × 10^5^ WT *C. albicans*, 72 h post-infection stained for CD3 and antibodies specific for the cleaved activated form of either caspase-3 or caspase-8 (*n* = 4 mice per group). **f** Immunofluorescent micrographs from inguinal lymph nodes of naïve WT mice or infected with 5 × 10^5^ WT *C. albicans* and pre-treated with control or anti-SIGNR1 antibodies, 72 h post-infection. Stained for TUNEL and CD3 at lower magnification (upper row, scale bars 100 μm) and higher magnification (bottom row, scale bars 25 μm) (*n* = 4 mice per group). **g** Quantification of TUNEL + cells per field of view (FOV) in inguinal lymph nodes from **f**. Three FOVs per mouse and three mice per group. **h**, **i** Cell-free DNA (**h**) and histone H3 (**i**) in the plasma of WT mice pre-treated with control or anti-SIGNR1 antibodies and infected with 5 × 10^5^ WT *C. albicans*, 72 h post-infection (IgG *n* = 4 and anti-SIGNR1 *n* = 5 mice). Statistical analysis by unpaired two-sided Mann–Whitney *t*-test for single comparison and two-way Anova for T cell subset analysis (*p < 0.05, **p < 0.01, ***p < 0.001, ****p < 0.0001).

The induction of cytokines in MZ macrophages raised the question of whether these cells were undergoing pyroptotic cell death. Hence, we stained infected spleens with terminal deoxynucleotidyl transferase dUTP nick end labelling (TUNEL). We did not detect substantial TUNEL signal in MZ macrophages but there was extensive death of cells inside the white pulp of infected control-treated mice that was suppressed by inhibition of SIGNR1 (Fig. 4a, Supplementary Fig. 6a). Staining with T cell markers demonstrated that the dying cells were predominately T cells that were positive for CD4, CD3 and TCRβ (Fig. 4a, b, Supplementary Fig. 6a). Interestingly, a substantial number of TUNEL^+^ T cells could be found outside germinal centers (Fig. 4a, c, Supplementary Fig. 6a). We also confirmed the loss of T cells in the spleen by flow cytometry which demonstrated that cell death impacted T cells non-specifically and afflicted all Vβ-chain populations (Fig. 4d). Staining these splenic samples with antibodies against the activated forms of caspase-3 and caspase-8 confirmed that T cells were dying via apoptosis (Fig. 4e).

We also detected a SIGNR1-dependent increase in T cell death in the inguinal lymph nodes and the thymus although the latter was not statistically significant (Fig. 4f, g, Supplementary Fig. 6b). Interestingly, *C. albicans* was absent from lymph nodes and the thymus, indicating that T cell death was driven by systemic mediators (Supplementary Fig. 6c). Cell death also occurred in the kidneys and was significantly reduced in SIGNR1-blocked mice. However, dying kidney cells were not T cells as TUNEL positive cells were not positive for CD3 (Supplementary Fig. 6d). Given the extent of cell death, we examined whether SIGNR1 inhibition affected the release of systemic cell-free chromatin. DNA and histones were prominent in the plasma of infected IgG-treated mice but were absent in samples from anti-SIGNR1-treated mice (Fig. 4h, i). Collectively, these data demonstrate that SIGNR1 regulates T cell death and the release of extracellular chromatin in the circulation.

### T cells are required for circulating chromatin release

**Fig. 5:**
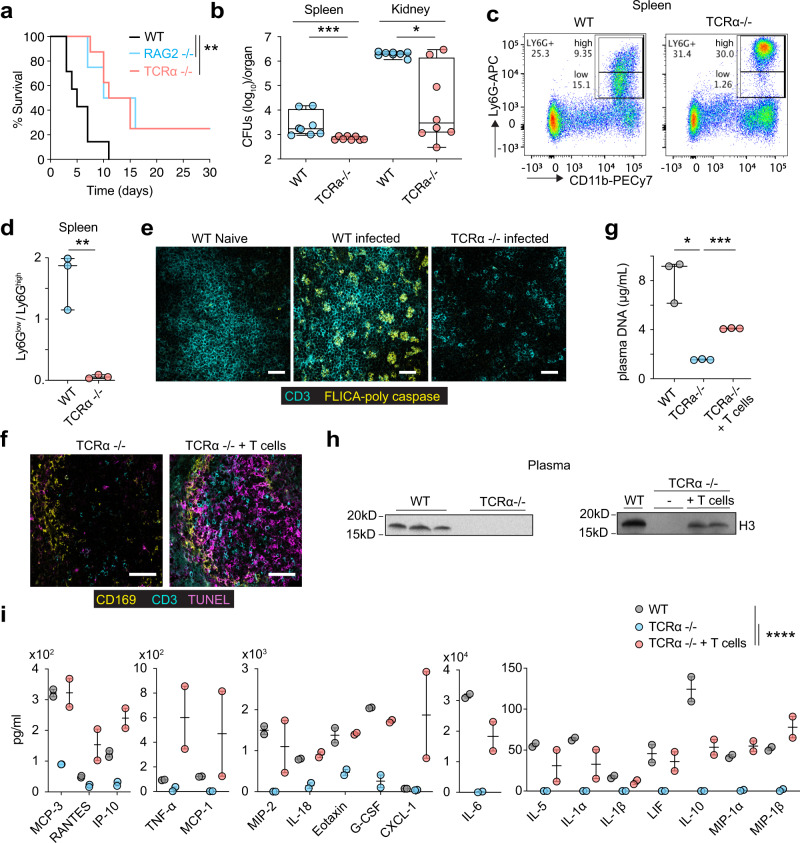
T cell death and circulating chromatin release regulate neutrophil alterations and inflammation. **a** Survival curves of WT, TCRα or RAG2-deficient mice infected with 5 × 10^5 ^*C. albicans* (*n* = 5, *n* = 8 and *n* = 4 mice per group respectively). **b** Colony forming units in the spleens and kidneys of WT and TCRα-deficient mice infected with 5 × 10^5 ^*C. albicans*, 72 h post infection. Representative of two experimental replicates with 8 mice per group. **c** Representative flow cytometric analysis of Ly6G^low^ and Ly6G^high^ neutrophils in the spleen of WT and TCRα-deficient mice infected with 5 × 10^5^ WT *C. albicans* and assessed as Live CD3^−^ CD19^−^ CD11b^+^ Ly6G^high^/Ly6G^low^ 72 h post-infection. (WT *n* = 3 and TCRa-deficient *n* = 3 animals). Panel: Live/Dead blue, CD3/CD19 PerCP-Cy5.5, CD11b-PECy7, Ly6G-APC. **d** Ly6G^low^/Ly6G^high^ neutrophil ratios in individual mice from (**c**). **e** Immunofluorescence micrographs from the spleens of naive or infected WT or TCRα-deficient mice infected with 5 × 10^5^ WT *C. albicans*, 72 h post-infection and stained for CD3 and poly-caspase enzyme activity performed with a FLICA poly-caspase activity assay (*n* = 3 animals per group and two independent experiments). Scale bars: 50 μm. **f** Immunofluorescence micrographs from the spleens of TCRα-deficient mice alone or after adoptive T cell transfer 48 h prior to infection and infected with 5 × 10^5^ WT *C. albicans*, 72 h post-infection (*n* = 3 mice per group and two independent experiments). Scale bar: 50 μm**. g**–**i** Cell-free DNA (**g**), western immunoblotting for histone H3 (**h**), and plasma cytokines and chemokines measured by multiplex immunoassay (**i**) of mice in **f**. Results from two independent experiments. Statistical analysis by unpaired two-sided Mann–Whitney *t*-test for single comparisons, two-tailed Log-rank (Mantel–Cox) test for survival analysis and two-way Anova for the cytokine array (*p < 0.05, **p < 0.01, ***p < 0.001, ****p < 0.0001).

To evaluate whether T cell death was pathogenic we examined the survival of TCRα-deficient mice that lack αβ-T cells and RAG2-deficient mice that lack T cells and B cells. Both strains exhibited improved survival upon systemic fungal challenge and carried a lower fungal load in their spleen and kidneys 3 days post-infection (Fig. 5a, b). Moreover, infected TCRα-deficient mice maintained Ly6G^high^ neutrophils in the spleen indicating that T cells are implicated in neutrophil dysfunction (Fig. 5c, d). Staining with a FLICA poly-caspase activity assay further confirmed the elevated incidence of cell death in T cells and was absent in the spleen of T cell-deficient mice (Fig. 5e). Moreover, TUNEL staining was absent from the spleens of infected TCRα-deficient mice and adoptive transfer of T cells restored cell death in the spleen white pulp (Fig. 5f). Likewise, circulating chromatin was absent in the plasma of TCRα knockout animals and could be restored by adoptive transfer of T cells (Fig. 5g, h). Furthermore, T cell deficiency led to a reduction in plasma cytokines, including IL-6 and G-CSF (Fig. 5i). Together, these observations indicated that dying T cells regulate the release of circulating chromatin, cytokine induction and alterations in neutrophil populations.

### MPO and T-cell death are relevant in bacterial sepsis

**Fig. 6:**
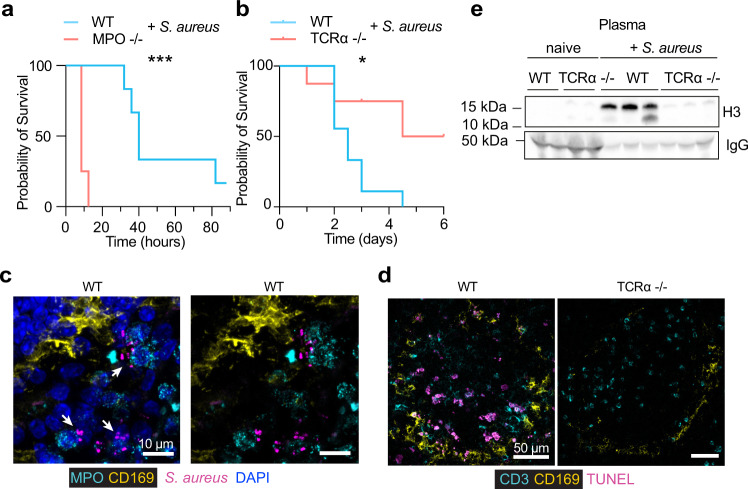
T cell death regulates pathology in bacterial sepsis. **a**, **b** Survival of WT, MPO and TCRα-deficient mice infected intravenously with 1 × 10^6^ WT *S. aureus* (*n* = 8 mice per group). **c**, **d** Confocal immunofluorescence micrographs from infected WT and TCRα-deficient mice stained for **c** *S. aureus*, DAPI, MPO and CD169 or **d** CD3, CD169 and TUNEL (*n* = 3 mice per group and two independent experiments). Scale bars 50 μm. **e** Western immunoblotting for histone H3 and IgG in the plasma of *S.* aureus-infected WT and TCRα-deficient mice 72 h post-infection (*n* = 3 mice per group). Statistical analysis by two-tailed Log-rank (Mantel–Cox) test for survival analysis (*p < 0.05, **p < 0.01, ***p < 0.001, ****p < 0.0001).

To examine whether these mechanisms are relevant during bacterial sepsis, we infected MPO and T cell-deficient animals systemically with *Staphylococcus aureus*. The results mirrored the systemic candidiasis experiments. MPO deficiency accelerated the onset of severe symptoms whereas αβ T cell deficiency delayed it (Fig. 6a, b). *S. aureus* colonization increased from 24 to 72 h post-infection, was not restricted to the MZ and could be detected throughout the red pulp but not the white pulp (Fig. 6c, Supplementary Fig. 7). T cell death was detectable in the spleens of *S. aureus*-infected mice and TCRα-deficiency decreased TUNEL staining in the spleen and eliminated cell-free histones from the circulation (Fig. 6d, e). Hence, T cell-death dependent histone release appeared to be relevant in systemic bacterial infection.

### Cell death accelerates alterations in neutrophil populations

**Fig. 7:**
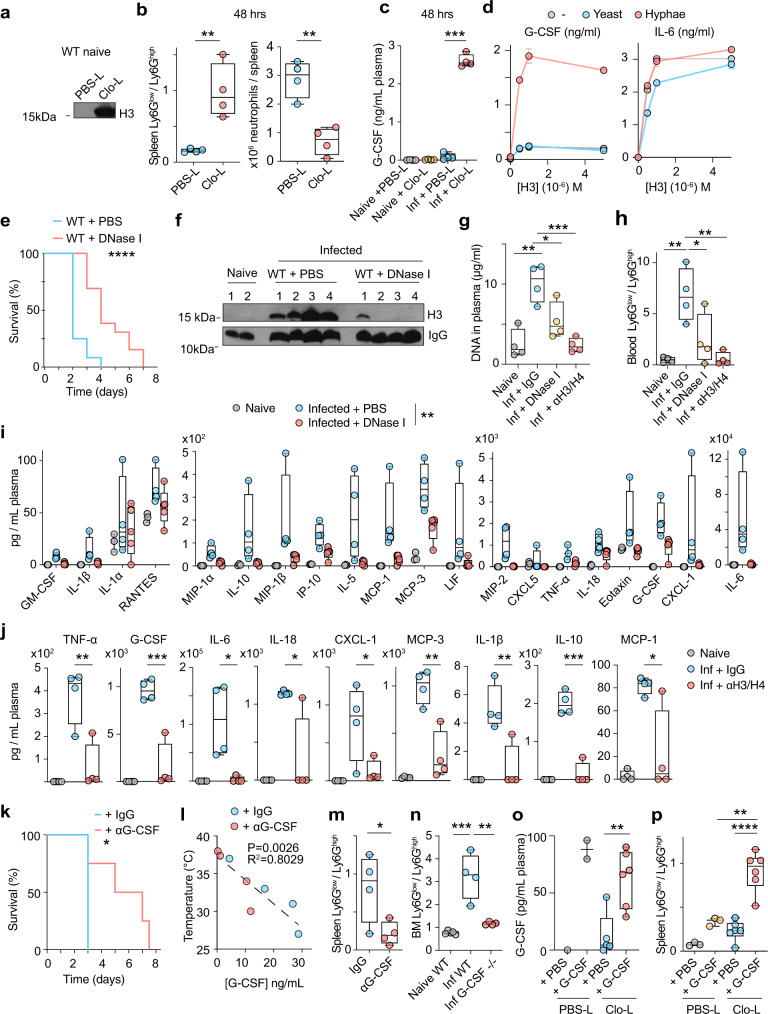
Extracellular chromatin and G-CSF alter neutrophil populations. **a** Western immunoblotting for plasma histone H3 in naïve WT mice 24 h after intravenous injection of PBS-liposomes (PBS-L) or clodronate-liposomes (Clo-L). **b**, **c** Ly6G^low^/Ly6G^high^ ratios (left panel) and total neutrophils (right panel) in the spleens (**b**) and plasma G-CSF concentrations assessed by simplex immunoassay (**c**) from naïve or infected WT mice with 1 × 10^5^ WT *C. albicans*, pre-treated with PBS-L or Clo-L, 48 h post-infection (*n* = 4 mice per group). Average and SD. **d** G-CSF (left) and IL-6 (right) protein production in WT BM-derived macrophages alone (grey) or in the presence of heat-inactivated yeast (blue) or hyphae (red) alone or in combination with increasing concentrations of recombinant histone H3. Average and SD from two technical replicates per condition. Representative of three independent experiments. **e** Survival of WT mice pre-treated with PBS (blue) or DNase I (red) and infected with 5 × 10^5^ WT *C. albicans*. Representative of 3 individual experiments with 6 (PBS) and 12 (DNase I-treated) mice per group. **f–j** Histone H3 (**f**), DNA levels (**g**) neutrophil Ly6G^low^/Ly6G^high^ ratios (**h**) and cytokines and chemokines (**i**, **j**) in the plasma of naïve or infected WT mice with 5 × 10^5^ WT *C. albicans*, pre-treated with either IgG control antibody, anti-H3 and anti-H4 antibodies, or DNase I, analysed 72 h post-infection (naïve *n* = 3 and infected *n* = 5 mice per group and two independent experiments). **k** Survival of WT mice infected with 5 × 10^5^ WT *C. albicans* and treated daily with control (blue) or anti-G-CSF (red) antibodies starting at 24 h post-infection (*n* = 4 mice per group and two independent experiments). **l**, **m** Correlation between plasma G-CSF concentrations and body temperature (**l**) and Ly6G^low^/Ly6G^high^ ratios in splenic neutrophils (**m**) at 72 h post-infection in infected WT mice treated with control (blue) or anti-G-CSF antibody (red) (*n* = 4 mice per group). **n** Ly6G^low^/Ly6G^high^ BM neutrophil ratio from naïve WT and G-CSF-deficient FVB/NJ mice or infected with 1 × 10^4^ or 1 × 10^3^ WT *C. albicans* respectively, 72 h post-infection (*n* = 4 mice per condition and two independent experiments). **o**, **p** Plasma G-CSF concentrations (**o**) and Ly6G^low^/Ly6G^high^ ratios (**p**) in splenic neutrophils from naïve mice treated with PBS-L or Clo-L, subsequently injected with PBS or recombinant G-CSF (rG-CSF) after 24 h and assessed 48 h later (PBS-L *n* = 3 and Clo-L *n* = 6 (Clo-L) mice per group, representative of two experiments). Panel: LD Blue, CD3/CD19 PerCP-Cy5.5, CD11b-PECy7, Ly6G-APC. Statistical analysis by unpaired two-sided Mann–Whitney *t*-test for single comparison, two-sided Log-rank (Mantel–Cox) test for survival analysis and two-way Anova for cytokine array with multiple comparisons (*p < 0.05, **p < 0.01, ***p < 0.001, ****p < 0.0001).

Next, we investigated whether cell death plays a role in cytokine induction and neutrophil dysfunction. Macrophage depletion experiments using Clo-L administration induced high concentrations of circulating chromatin in the absence of infection but did not cause pathology (Fig. 7a). We hypothesized that the phenotype we observed in infected Clo-L-treated mice was not only caused by a reduction in macrophages but could also be accelerated by pre-existing chromatin release. Consistent with this hypothesis, Clo-L treatment accelerated the emergence of Ly6G^low^ neutrophils and the reduction in total splenic neutrophil numbers upon infection (Fig. 7b, Supplementary Fig. 8a). Therefore, cell death and circulating histones alone were not sufficient to induce pathology in the absence of infection but accelerated cytokine induction and neutrophil population alterations upon infection.

### Circulating chromatin and fungi induce G-CSF

We hypothesized that G-CSF might be implicated in the dysregulation of neutrophil populations as the emergence of Ly6G^low^ neutrophils coincided with the upregulation of G-CSF in our experiments and the cytokine has been linked to the presence of immature neutrophils in murine cancer models^39^. First, we examined whether Clo-L affected G-CSF induction in naive and infected mice. Clo-L alone in the absence of infection had little effect on the plasma concentrations of G-CSF, but Clo-L pre-treatment amplified plasma G-CSF induction upon fungal challenge (Fig. 7c). These data suggested that circulating histones and *C. albicans* could synergize in vivo to induce G-CSF. To test this hypothesis, we incubated BM-derived macrophages with recombinant histone H3 alone or in combination with heat-inactivated yeast or hyphae. Histones or hyphae alone were weak G-CSF inducers, but the two signals acted synergistically to induce high G-CSF levels (Fig. 7d). This synergy depended on the hyphal form, as yeast were unable to induce G-CSF in the presence of histone H3. By contrast, histone H3 was sufficient to induce IL-6 and fungi had no effect on the induction of this cytokine suggesting a divergence in the pathways that regulate IL-6 and G-CSF.

### Circulating chromatin and G-CSF are required to alter neutrophil populations

To test the impact of histones on neutrophils we sought an approach that would clear circulating chromatin enzymatically. We previously found that proteases and endonucleases synergize in chromatin clearance in the sputum of patients with cystic fibrosis^40^. Therefore, we tested whether intraperitoneal administration of DNase I could promote chromatin clearance in the plasma that contains serum proteases^6^. We also treated mice with histone-blocking antibodies. DNase I administration delayed sepsis in infected mice (Fig. 7e) and cleared free circulating DNA and histones (Fig. 7f, g). Moreover, mice treated with either DNase I or histone-blocking antibodies maintained high circulating Ly6G^high^ neutrophil populations (Fig. 7h, Supplementary Fig. 8b) and exhibited lower plasma cytokine levels, including G-CSF and IL-6 (Fig. 7i, j, Supplementary Fig. 9). Histone blockade was even more effective than DNAse I treatment in suppressing cytokine induction and a high Ly6G^low^/Ly6G^high^ ratio.

The link between cell death-derived chromatin and G-CSF led us to investigate whether G-CSF played a role in the dysregulation of neutrophils during systemic candidiasis. G-CSF receptor (G-CSFR)-deficient mice are more susceptible to systemic *C. albicans* challenge despite exhibiting effective emergency granulopoiesis and neutrophil mobilization from the BM^41^. However, in our experiments, G-CSF correlated with neutrophil dysfunction and was upregulated by SIGNR1 and circulating chromatin. Therefore, we hypothesized that prolonged exposure to G-CSF may promote pathology during the course of infection. To test this hypothesis, we neutralized G-CSF by daily administration of a blocking antibody, starting at 24–48 h post-infection, in order to avoid interfering with any protective functions of G-CSF during the early phases of the infection. Late anti-G-CSF treatment resulted in a significant delay in the onset of severe symptoms (Fig. 7k). Moreover, elevated G-CSF plasma concentrations correlated with a decrease in body temperature (Fig. 7l) and G-CSF neutralization suppressed the emergence of Ly6G^low^ neutrophils (Fig. 7m, Supplementary Fig. 10a). We also infected G-CSF-deficient mice with *C. albicans* and examined the effect on neutrophil populations. Due to peripheral neutropenia, these mice are more susceptible to *C. albicans* systemic infection and therefore we infected with a low 1 × 10^3 ^*C. albicans* inoculum which induced severe symptoms 2–3 days post-infection. Given that G-CSF elicits neutrophil egress from the BM we analysed neutrophils in the BM to ensure that any measured differences were not due to lack of neutrophil mobilization. Notably, the Ly6G^low^/Ly6G^high^ neutrophil ratio in infected symptomatic G-CSF-deficient mice remained low suggesting that G-CSF is required for the observed alterations in neutrophil populations (Fig. 7n).

### Cell death and G-CSF are sufficient to alter neutrophil populations in the absence of infection

The administration of G-CSF in the absence of infection is not known to cause deleterious effects on granulopoiesis. However, G-CSF is required, but not sufficient for the emergence of minor populations of low-density granulocytes in murine cancer models^39^. Therefore, we hypothesized that G-CSF may synergize with cell death-derived chromatin in promoting neutrophil dysfunction. Moreover, we sought conditions where we could decouple the emergence of Ly6G^low^ neutrophils from infection. To test this hypothesis, we first treated mice with PBS liposomes (PBS-L) or Clo-L and subsequently administered recombinant G-CSF after 24 h. We assessed splenic neutrophil populations by flow cytometry 48 h later. At that time, PBS-L and Clo-L-treated groups that had received G-CSF had comparable G-CSF concentrations in their blood (Fig. 7o). However, while G-CSF administration in PBS-L-treated mice did not alter the neutrophil ratio substantially, the combination of Clo-L and G-CSF induced a Ly6G^low^/Ly6G^high^ neutrophil ratio that was comparable to that observed in infected symptomatic mice (Fig. 7p, Supplementary Fig. 10b). Moreover, pre-treating mice with G-CSF 24 h prior to infection delayed the onset of sepsis whereas late administration had no beneficial effects, indicating that boosting granulopoiesis prior to infection is beneficial, but increasing G-CSF levels at later stages provides no benefit (Supplementary Fig. 10c). These results were also consistent with the idea that G-CSF may play distinct roles in the presence and in the absence of circulating chromatin. Together these data indicated that in addition to the ability of histones to induce G-CSF in the presence of systemic infection, chromatin also acts downstream with G-CSF to interfere with the antimicrobial fitness of neutrophil populations.

### The transcriptional signature of Ly6G^low^ neutrophils resembles immature populations and is regulated by T cells

**Fig. 8:**
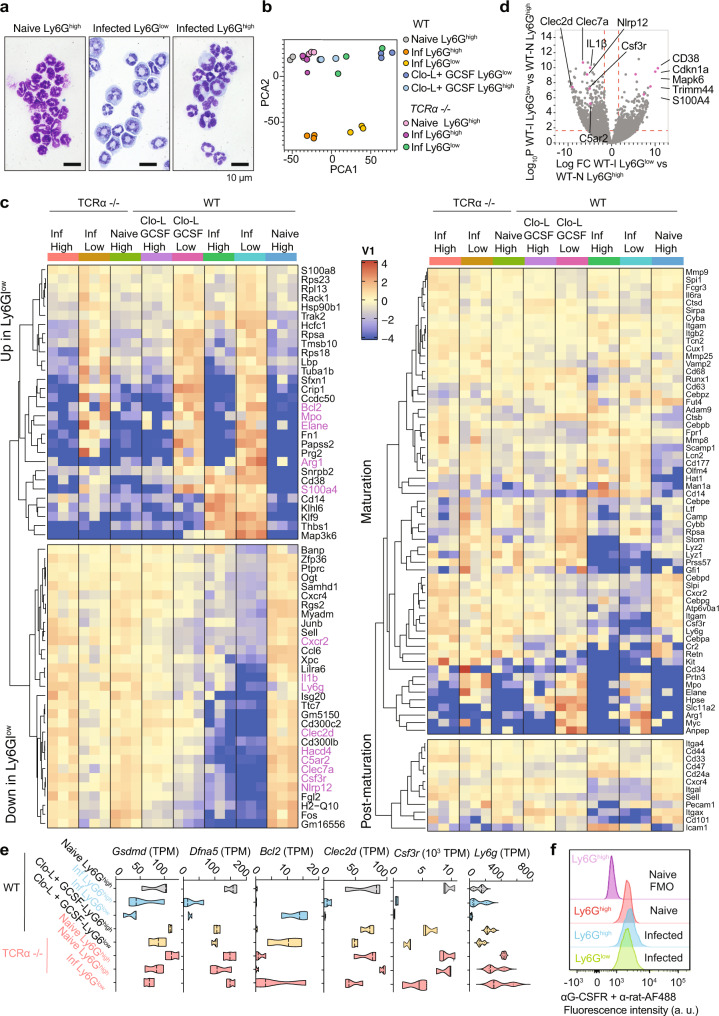
Transcriptomic profiling of neutrophils during infection and sterile challenge. **a** Giemsa-Wright staining of Ly6G^low^ and Ly6G^high^ neutrophils from WT naïve mice or infected with 5 × 10^5^ WT *C. albicans*, 72 h post-infection, that were cyto-spun following sorting by flow cytometry. Sorted cells were Live CD3^−^CD19^−^CD11b^+^Ly6G^high/low^. Panel: DAPI, CD3/ CD19-FITC, CD11b-PE, Ly6G-APC. Representative images of 3–4 mice per group. Scale bar: 10 μm. **b** Principal component analysis of transcripts from sorted Ly6G^low^ and Ly6G^high^ neutrophils isolated from WT and TCRα-deficient mice, either naïve or infected with 5 × 10^5^ WT *C. albicans* (48 h post-infection) and naïve WT mice treated with Clo-L and recombinant G-CSF (*n* = 3 mice per group). **c** Expression profiling heatmaps for the (left) top genes that are differentially expressed and (right) classical maturation and post-maturation markers in neutrophils from all groups in **b** (*n* = 3 mice per group). **d** Volcano plot comparing expression profiling of flow cytometric sorted Ly6G^high^ and Ly6G^low^ neutrophils from WT naïve and infected mice respectively, 48 h post-infection. **e** Transcript reads per million (TPM) of *Gsdmd*, *Dfna5*, *Bcl2*, *Clec2d*, *Csf3r* and *Ly6g*. **f** Flow cytometric analysis of G-CSFR protein surface expression in Ly6G^high^ neutrophils from naïve mice and Ly6G^high^ and Ly6G^low^ neutrophils from mice infected with 5 × 10^5^ WT *C. albicans*, 72–96 h post-infection. Includes an FMO control containing only secondary antibody. Panel: LD blue, CD3/CD19 PerCP-Cy5.5, CD11b-PECy7, Ly6G-APC, anti-GCSFR +anti-rat-AF488 (naïve *n* = 5 and infected *n* = 4 mice per group). Statistical analysis by unpaired two-sided Mann–Whitney *t*-test (*p < 0.05, **p < 0.01, ***p < 0.001, ****p < 0.0001).

To better understand the changes in neutrophil populations during sepsis and the impact of T cell death, we profiled neutrophils populations. Wright-Giemsa staining indicated that FACS-sorted Ly6G^low^ neutrophils from symptomatic mice, comprised a mixed population of mature lobulated and morphologically immature band cells (Fig. 8a). To compare these cells at the molecular level, we conducted RNA sequencing analysis of Ly6G^high^ and Ly6G^low^ neutrophils sorted by flow cytometry from the spleens of either naïve or infected WT or TCRα-deficient animals and uninfected WT mice receiving exogenous G-CSF and Clo-L. PCA analysis indicated that Ly6G^high^ and Ly6G^low^ populations from infected WT symptomatic mice were distinct from one another but also clustered differently from Ly6G^high^ neutrophils isolated from infected asymptomatic TCRα-deficient animals or naïve WT mice (Fig. 8b). Moreover, Ly6G^low^ neutrophils in G-CSF and Clo-L treated mice were distinct from naïve Ly6G^high^ cells but also exhibited variations compared to Ly6G^low^ neutrophils found in infected symptomatic mice. Next, we compared these populations based on the major gene expression differences between septic Ly6G^low^ and Ly6G^high^ cells across the entire dataset. Ly6G^low^ neutrophils from infected WT mice had a distinct signature in comparison to Ly6G^high^ populations but also when compared to Ly6G^low^ neutrophils in infected asymptomatic TCRα-deficient mice (Fig. 8c). Given the asymptomatic state, the TCRα-deficient Ly6G^low^ cells comprised a “healthy” immature population under a state of infection. TCRα-deficient mice exhibited a Ly6G^high^ population that had similar gene expression patterns to Ly6G^high^ populations from naïve WT mice. Moreover, while WT infected Ly6G^low^ cells exhibited similarities to Ly6G^low^ cells from TCRα-deficient mice, they also bore distinct transcriptional differences that set them apart from immature cells associated with an infected asymptomatic state.

There was a cluster of genes with similar trends amongst the Ly6G^low^ cells isolated from uninfected G-CSF/Clo-L-treated-mice and the Ly6G^low^ neutrophils derived from infected symptomatic WT mice, supporting the hypothesis that G-CSF and chromatin promote shifts in neutrophil populations during infection. We also examined the expression of genes that are associated with the different stages of neutrophil maturation and found that Ly6G^low^ neutrophils exhibited reduced expression in several maturation and post-maturation genes (Fig. 8c). Comparison of infected WT Ly6G^low^ with Ly6G^low^ neutrophils from TCRα-deficient mice also highlighted changes in a number of transcripts that included *Cxcr2*, *CD14*, *Itgam*, *Csf3r*, *CD34*, *Cr2* and *Arg1* that distinguished this population from TCRα-deficient Ly6G^low^ cells. Infected WT Ly6G^low^ neutrophils also lacked transcripts for C-type-lectin-receptors *Clec2d and Clec7a* that recognize histones and fungal pathogens respectively, as well as the complement receptor C5ar2, two Nod-like receptors and IL-1β, that could further compromise their antifungal activity (Fig. 8c, d, e). Notably, while *Ly6g* transcripts were downregulated in Ly6G^low^ cells and to a lesser degree in Ly6G^high^ neutrophils from infected WT spleens, transcripts levels were high in both populations isolated from infected TCRα-deficient mice, although this difference was not reflected at the protein level (Fig. 8e). Infected WT Ly6G^low^ cells exhibited additional adaptations that distinguished them from TCRα-deficient Ly6G^low^ neutrophils such as low levels of *Cxcr2*, *Gsdmd* and *Dfna5* (gasdermin D and E) and higher *Bcl2* transcript levels which may increase cell survival (Fig. 8e). Moreover, transcripts for the G-CSF receptor *Csf3r* were downregulated in Ly6G^high^ and Ly6G^low^ neutrophils from infected mice or uninfected mice treated with G-CSF and Clo-L but not in cells from naïve or TCRα-deficient mice. The downregulation of G-CSFR mRNA could result from a negative feedback response to sustained G-CSF signalling. However, the G-CSFR protein could still be detected on the surface of Ly6G^high^ and Ly6G^low^ neutrophils from infected WT mice at comparable intensity to Ly6G^high^ cells from naïve WT mice indicating that these cells could still sense G-CSF (Fig. 8f, Supplementary Fig. 10d). These data confirmed that peripheral neutrophil populations in symptomatic mice were predominately immature cells that were molecularly distinct from peripheral mature and immature neutrophils found in infected mice lacking T cells, indicating that additional changes occur with disease severity.

### T cell-derived histones eliminate mature Ly6G^high^ neutrophils but not Ly6G^low^ neutrophils in the bone marrow

**Fig. 9:**
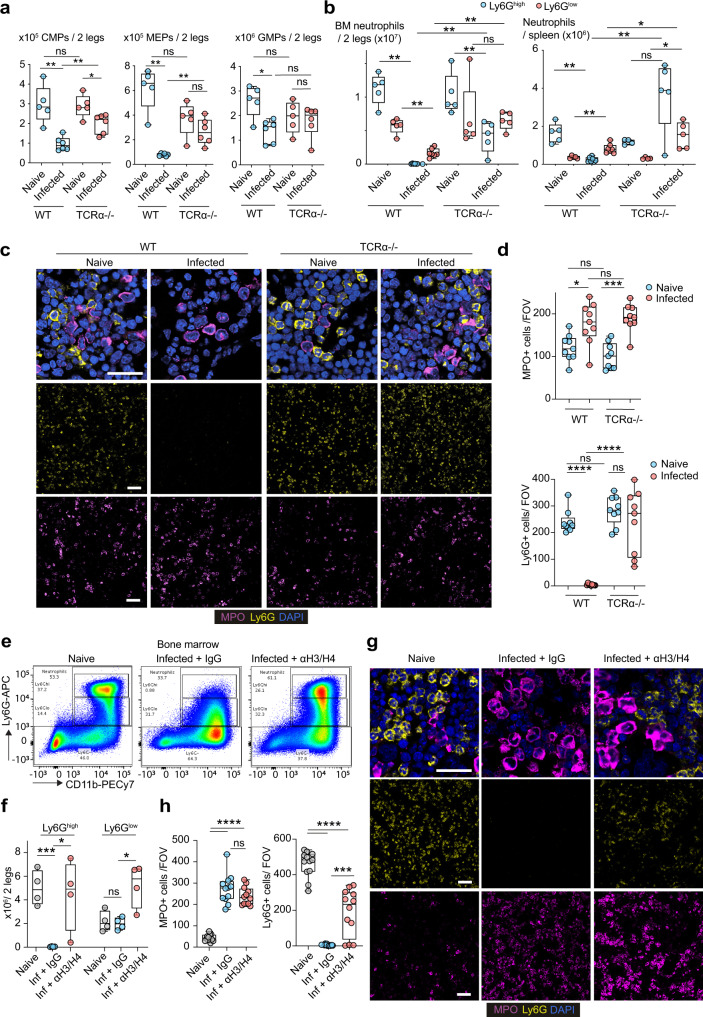
Histones deplete mature Ly6G^high^ neutrophils in the bone marrow. **a** Flow cytometry analysis of myelopoietic progenitors and mature Ly6G^high^ neutrophils in the BMs of naive and infected WT and TCRα-deficient animals. Total numbers of common myeloid progenitors (CMP), megakaryocyte-erythrocyte progenitors (MEP) and granulocyte-monocyte progenitors (GMP) are shown. HSCs: Lineage (CD3, CD4, CD8, GR1, B220 and TER119)^−^ c-KIT^+^ Sca-1^−^. MEPs: CD16/32^low/-^. CMPs: CD16/32^intermediate^, CD34^+^. GMPs: CD16/32^high^. Panel: CD3/CD4/CD8/GR1/B220/TER119-biotin-streptavidinBV421, c-KIT-BV711, Sca-1-BUV395, CD16/32-PE, CD34-FITC. *n* = 5 (naïve) and 6 (infected) mice per group. **b** Total Ly6G^low^ and Ly6G^high^ neutrophils in the BM and spleens of naïve and infected WT and TCRα-deficient mice. Panel: Live/Dead blue, CD3/CD19 PerCP-Cy5.5, CD11b-PECy7, Ly6G-APC (naïve *n* = 5 and infected *n* = 6 mice per group). **c** Immunofluorescence micrographs from the BM of naïve and infected WT and TCRα-deficient mice 3 days post infection stained for MPO, Ly6G and DAPI (*n* = 3 mice per group and 2 independent experiments). Scale bars: 50 μm. **d** Number of MPO^+^ and Ly6G^+^ neutrophils per FOV (*n* = 3 mice per condition and 3 FOV per mouse). **e** Representative flow cytometry of neutrophils in the BM of naïve and infected WT mice pre-treated with a control antibody or antibodies against histones H3 and H4. Panel: LD blue, CD3/CD19 PerCP-Cy5.5, CD11b-PECy7, Ly6G-APC (*n* = 4 mice per group 2–3 days post-infection and two independent experiments). **f** Total Ly6G^low^ and Ly6G^high^ neutrophils in the BM of animals from **e**. **g** Immunofluorescence micrographs from the BM of naïve and infected WT mice pre-treated with an isotype IgG or antibodies against histones H3 and H4, 3 days post infection stained for MPO, Ly6G and DAPI. Scale bars: 50 μm. **h** Quantification of the number of mature (Ly6G^+^) and immature (MPO^+^) cells per FOV in **g** (*n* = 4 mice per group and 3 FOVs per mouse). Statistical analysis by unpaired two-sided Mann–Whitney *t*-test (*p < 0.05, **p < 0.01, ***p < 0.001, ****p < 0.0001).

To explore the underlying mechanism behind the change in neutrophil populations, we examined the total numbers of neutrophils and their progenitors in the BM. Symptomatic infection in WT mice was accompanied by decreases in common myeloid progenitors (CMPs) as well as their derivative granulocyte-monocyte progenitors (GMPs) and megakaryocyte-erythrocyte progenitors (MEPs) (Fig. 9a). The size and ratio of these populations remained largely unaffected by infection in asymptomatic TCRα-deficient mice with only CMPs exhibiting a slight reduction (Supplementary Fig. 11a). By contrast CMP numbers were reduced by 3-fold in infected WT mice compared to naïve WT controls. The number of CMPs and MEPs was also reduced in infected WT mice in comparison to TCRα-deficient mice, whereas there was no difference between GMPs amongst the two groups. This was an important observation because there was a dramatic decrease in mature Ly6G^high^ neutrophils in the BM and the spleen of infected WT mice (Fig. 9b). Ly6G^low^ neutrophils were only reduced by ~50% which resulted in these cells becoming the predominant neutrophil population in the BM and in the periphery. By contrast, TCRα-deficient mice maintained a substantial mature Ly6G^high^ population and their Ly6G^low^ neutrophil numbers remained unchanged in the BM.

We took advantage in the relative difference between MPO and Ly6G expression levels in Ly6G^low^ and Ly6G^high^ neutrophils as determined by RNAseq expression analysis in order to distinguish these cells by immunofluorescence microscopy in the BM. Consistently, the MPO signal in immature Ly6G^low^ cells in the BM was high compared to mature Ly6G^high^ neutrophils which enabled the specific detection of immature neutrophils by immunofluorescence microscopy. Similarly, Ly6G staining detected specifically mature Ly6G^high^ neutrophils. As in the flow cytometry analysis, Ly6G^high^ neutrophils were completely absent from the BM of infected WT mice but were still present in infected TCRα-deficient BM (Fig. 9c, d). These findings indicated that neutrophil population changes occurred predominately at the late maturation stage, rather than during early differentiation, with a profound loss of mature Ly6G^high^ neutrophils. Importantly, the infection impacted predominately mature neutrophil populations and to a much lesser degree immature granulocytes and their progenitors, since the BM of symptomatic WT and asymptomatic TCRα-deficient contained relatively equal numbers of GMPs and immature neutrophils.

To assess the role of histones in this process, we also examined the impact of histone-blocking antibodies on BM neutrophil populations. A cocktail of anti-H3 and anti-H4 antibodies maintained a significant presence of mature Ly6G^high^ neutrophils in the BM detected by flow cytometry (Fig. 9e, f) and by microscopy (Fig. 9g, h), indicating that histones play an important role in eliminating mature neutrophils from the BM.

### Histones and G-CSF alter neutrophil populations by modulating neutrophil lifespan

**Fig. 10:**
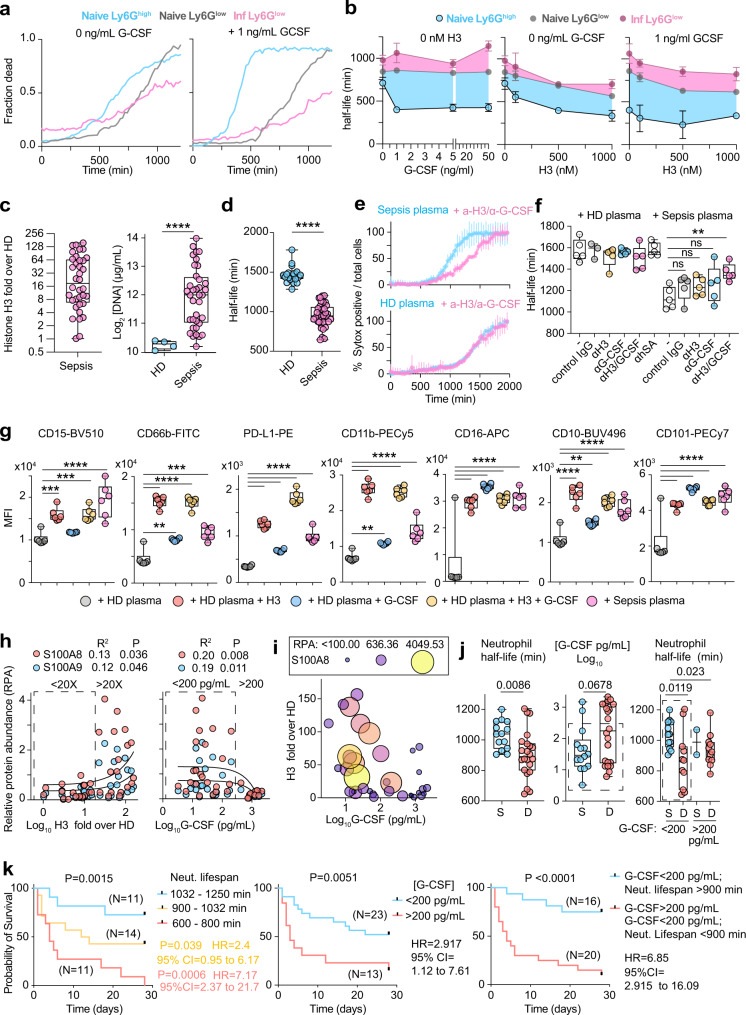
Histones and G-CSF shorten mature neutrophil lifespan selectively. **a** Representative neutrophil death time-courses of Ly6G^high^ (blue) and Ly6G^low^ (grey) BM neutrophils sorted from naïve WT mice and Ly6G^low^ cells (pink) from the BM of symptomatic WT mice infected with *C. albicans* in the absence or in the presence of 1 ng/mL G-CSF (Representative of 2 independent experiments. **b** Effect of recombinant histone H3 and G-CSF alone or in combination, on mature and immature neutrophil half-life curves for Ly6G^high^ (black) naive Ly6G^low^ (grey) and infected Ly6G^low^ (pink). Differences between curves are highlighted by coloured windows. Each point is the average half-life from three separate field measurements. **c** Fold increase over 4 healthy donors (HD) in histone H3 in the plasma of 36 human patients with bacterial sepsis, quantified from western immunoblots (left). DNA concentrations in the plasma of 4 HDs and 36 sepsis patients (right). **d** Half-life HD blood neutrophils incubated with plasmas from 22 HDs (blue) or 36 sepsis patients (pink). Representative of two independent experiments. **e** Representative cell death curves of HD neutrophils incubated with HD (lower) or sepsis patient plasma (upper) in the presence of a control or anti-histone H3 and anti-G-CSF antibodies. Average and SD for 4 FOV per condition. **f** Quantification of the half-life of HD neutrophils incubated with either HD (*n* = 5 donors) or sepsis patient (*n* = 5 donors) plasmas alone or in combination with anti-histone H3 or anti-G-CSF antibodies (3 FOV per donor). **g** Quantification of the mean fluorescence intensity (MFI) of surface markers in HD neutrophils incubated with HD or sepsis patient plasma alone or HD plasma supplemented with histone H3 or G-CSF, measured by flow cytometry. Each dot corresponds to a different plasma donor (*n* = 6). Panel: CD15-BV510, CD66b-FITC, PD-L1-PE, CD11b-PECy5, CD16-APC, CD10-BUV496, CD101-PECy7. Representative of two independent experiments. **h** Correlation between the fold-increase in sepsis patient plasma histone H3 (left plot) or G-CSF (right plot) and the relative abundance of S100A8 (red) and S100A9 (blue) measured by mass spectrometry. Boxes depict medium and high histone H3 containing plasmas. **i** Correlation between plasma G-CSF and histone H3 levels in sepsis patient plasmas. Dot sizes and colours are relative to plasma S100A8 levels. **j** Half-life of HD neutrophils incubated with plasmas from survivors (S) or deceased (D) sepsis patients (left), plasma G-CSF levels (middle) and neutrophil lifespan shortening activity in plasmas segregated into high (>200 pg/mL) and medium (dotted box, <200 pg/mL) G-CSF levels (right). **k** Survival curves of sepsis patients segregated into high (red), medium (yellow) and low (blue) neutrophil lifespan shortening activity (left), patients with high (red) and medium (blue) G-CSF levels and patients with medium G-CSF with low neutrophil lifespan shortening activity (blue) or high G-CSF and medium G-CSF with high neutrophil lifespan shortening activity (red) (right). Statistical analysis by simple linear regression, unpaired two-sided Mann–Whitney or parametric *t*-test and two-sided Log-rank (Mantel–Cox) test for survival analysis (*p < 0.05, **p < 0.01, ***p < 0.001, ****p < 0.0001).

To investigate how these signals could impact the abundance of distinct neutrophil populations, we sorted Ly6G^low^ and Ly6G^high^ neutrophils from the BM of naïve WT mice as well as Ly6G^low^ neutrophils from the BM of infected symptomatic WT mice and incubated them with varying concentrations of G-CSF and histone H3 alone, or in combination. We monitored cell death by time-lapse immunofluorescence microscopy over 1300 min and fitted the time-resolved cell death rates to measure neutrophil half-lives (Fig. 10a). Untreated mature Ly6G^high^ neutrophil populations from naïve mice had a slightly shorter lifespan than Ly6G^low^ cells from naïve mice, whereas Ly6G^low^ cells from infected mice exhibited the longest lifespan (Fig. 10a, b, Supplementary Fig. 11b, c). G-CSF treatment potently reduced the lifespan of Ly6G^high^ neutrophils but minimally affected naive Ly6G^low^ cells and extended the lifespan of Ly6G^low^ cells from infected mice, resulting in a large difference between the lifespans of Ly6G^high^ and Ly6G^low^ cells, particularly the ones isolated from infected symptomatic mice. On the other hand, recombinant histone H3 treatment decreased the lifespan of all three populations in a non-selective dose-dependent manner. A combination of modest concentrations of histone H3 (500 nM) and G-CSF (1 ng/mL) led to the largest decreased in mature neutrophil lifespan and the largest difference compared to the lifespan of Ly6G^low^ cells from infected mice. Therefore, G-CSF shifted neutrophil populations disproportionately in favour of immature neutrophils by selectively shortening the lifespan of mature cells and this activity could be potentiated by extracellular histones.

To investigate the clinical relevance of this mechanism in human sepsis we measured the levels of G-CSF and histones in the plasma of sepsis patients with early (<24 h) and severe (norepinephrine requirement > 0.4 μg/kg/min) bacterial septic shock^42^. We opted to examine a group of patients with bacterial infections because bacterial sepsis cases are more frequent than fungal-associated sepsis, and although the upstream mechanisms regulating the induction of these cues may vary, we sought to understand whether the signals played a general pathogenic role. Many patients exhibited elevated plasma histone H3 and DNA concentrations when compared to healthy donors (Fig. 10c, Supplementary Fig. 12a). Therefore, we incubated primary human neutrophils from the blood of healthy volunteers with 3% sepsis patient plasma and measured their lifespan over 2000 min. Sepsis plasma reduced mature human neutrophil lifespan by ~30% compared to healthy donor (HD) plasma (Fig. 10d). This decrease depended on G-CSF and histones since neutralising antibodies against histone H3 and G-CSF significantly blocked lifespan reduction by sepsis plasma (Fig. 10e, f). The long duration of these shortened lifespans was inconsistent with the immediate activation of cell death as cells persisted for 9 h before gradually starting to die. By contrast, triggers of apoptosis lead to rapid cell death^43^. We also observed changes in neutrophil surface markers analysed by flow cytometry after 1000 min of incubation with sepsis plasma, compared to neutrophils incubated with HD plasma. Sepsis plasma which lowered neutrophil half-life below 1000 min, or HD plasma complemented with recombinant histone H3 and G-CSF, increased the surface expression of CD11b, CD15, CD16, CD66b, CD10, CD101 and PD-L1 and these markers may enable the monitoring of lifespan modulation in patient blood (Fig. 10g, Supplementary Fig. 12b).

To assess the impact of this mechanism on sepsis severity, we analysed the proteomic profile of patient plasmas and searched for significant correlations between histones, G-CSF and plasma proteins. Interestingly, we found that histone H3 correlated with increased concentrations of cell-free plasma S100A8/9, which are proteins expressed in the cytosol of neutrophils and are released upon neutrophil death (Fig. 10h). Elevated plasma S100A8/9 in sepsis patients is associated with increased risk for mortality^43^. S100A8/9 levels were elevated in patients with intermediate G-CSF plasma concentrations but were suppressed in samples containing high G-CSF concentrations (Fig. 10h, i, Supplementary Fig. 12c). Separating samples into medium and high G-CSF and histone groups uncovered a significant increase in S100A8/9 levels in patients with intermediate G-CSF and high histone H3 plasma concentrations (Fig. 10i, Supplementary Fig. 12c). Consistently, histone H3 levels were also low in samples that contained high G-CSF levels (Fig. 10i, Supplementary Fig. 12d) which may reflect the complex mechanisms of synergy in G-CSF induction and potential feedback regulation of histone release.

Given these complexities we explored which parameters correlate with sepsis patient survival. The early baseline plasma of patients who did not survive exhibited a significantly higher neutrophil lifespan lowering activity compared to patients who survived (Fig. 10j). The deceased group plasmas also contained higher levels of G-CSF in a bimodal distribution that indicated that high G-CSF levels correlate with mortality, but intermediate G-CSF levels were ambiguous. However, neutrophil lifespan shortening activity could distinguish survivors from non-survivors in the group containing intermediate G-CSF levels (Fig. 10j). In contrast, the two groups exhibited similar plasma histone H3 levels (Supplementary Fig. 12e). We then explored whether there were different clusters of mortality rates associated with different ranges of G-CSF levels or neutrophil lifespan shortening activity. Patients segregated by high, intermediate and low neutrophil lifespan shortening activity or plasma G-CSF levels exhibited different survival probabilities that indicated that poor outcomes are associated with high plasma G-CSF levels and high neutrophil lifespan shortening activity (Fig. 10k). These results where further confirmed when we combined the two parameters by placing patients into a group that containing high and intermediate G-CSF levels with high neutrophil lifespan shortening activity, and a group that contained patients with medium G-CSF levels and low neutrophil lifespan shortening activity. The high G-CSF and neutrophil lifespan shortening activity group had a 6.85-fold higher mortality probability with a *P*-value of <0.0001 suggesting that these strategies could be employed to improve the early identification of high risk patients. Together these findings suggest that neutrophil lifespan shortening activity is associated with pathology and high mortality in patients with septic shock.

## Discussion

Our findings demonstrate that by controlling circulating pathogens captured by splenic macrophages, MPO plays a critical protective role during disseminated infection to attenuate a pathogenic hyper-inflammatory programme that promotes immune dysfunction. Hence, MPO deficiency can result in deficiency in NADPH oxidase activity with more detrimental consequences for immune defence. This phenomenon explains the rare but severe episodes of systemic fungal infection in patients with complete MPO deficiency and is consistent with one of our MPO-deficient patients being misdiagnosed with CGD in the acute phase of infection due to the lack of superoxide production^28^. Furthermore, fungal capture by SIGNR1 progressively compromises the effectiveness of MPO by eliminating mature neutrophils that potently produce upstream oxidants to fuel the MPO enzyme. These events highlight the importance of maintaining immune barrier integrity in the spleen whose compromise produces compounds that disrupt immune defence in other organs.

During the initial phase of systemic infection, mature neutrophils control fungi captured by SIGNR1^+^ macrophages via MPO-derived ROS. However, progressive fungal colonization of the MZ promotes T cell death and the release of extracellular chromatin that synergizes with hyphae to induce G-CSF in CD169^+^ MZ macrophages. Sustained G-CSF production and T cell-derived chromatin compromise antimicrobial function by eliminating competent neutrophils. The remaining immature cells are unable to produce a potent hydrogen peroxide burst to fuel the MPO enzyme (Supplementary Fig. 13). Therefore, fungi exploit fungal capture by MZ macrophages to evade neutrophils and subvert their antimicrobial effector function by eliminating mature neutrophils via the destruction of T cells. This mechanism links immune dysfunction in neutrophils and T cells. Neutrophils attenuate T cell death via microbial control and T cell apoptosis promotes the release of pathogenic factors that deplete mature immunocompetent neutrophils. This model is consistent with the notion that while the spleen is essential for the effective clearance of circulating pathogens, pathogens can actively exploit the spleen’s microbe capture mechanisms as a “trojan horse” to neutralize the spleen’s antimicrobial function. In this scenario SIGNR1 and MPO play competing roles, with MPO being critical to protect the spleen from infection and SIGNR1-mediated capture eliciting a pathogenic programme that undermines MPO effector mechanisms. Similarly, while SIGNR1^+^ and CD169^+^ macrophages play beneficial roles in clearing blood-borne bacterial and viral infections^44–46^, retroviruses can hijack this process to infect T cells^47^. MPO protects the spleen and other organs against bacteria and fungi although its contribution depends on the pathogen species^29,48^. The colonization pattern of *S. aureus* in the spleen suggests that it is captured by red pulp macrophages, involving different receptors than SIGNR1. However, the downstream histone-mediated pathogenic programme appears to be implicated in both bacterial and fungal infections.

These findings shed light on the mechanisms that regulate the release of circulating histones during sepsis. Neutrophils are not a major source of circulating histones as shown previously by neutrophil depletion experiments^49^. Instead, we report a cell population whose depletion abrogates plasma chromatin release suggesting that dying T cells are key regulators and the most likely source of cell-free chromatin in the circulation. While the mechanisms that drive T cell death in the spleen remain undefined, this process may depend on signals from CD169^+^ macrophages as demonstrated in other models via the action of monocyte-derived Fas ligand^18^. Consistently, the activation of caspase-8 and caspase-3 and chromatin fragmentation are all indicative of T cell apoptosis. However, death of other splenocytes is also sufficient to promote systemic chromatin release as demonstrated by macrophage depletion experiments with Clo-L in uninfected mice which indicate that circulating chromatin alone is not sufficient to cause acute pathology in the absence of infection. Prior studies that demonstrated direct lethality upon administration of recombinant histone resulted in blood concentrations that were likely to be 100-fold higher than what we detected in naïve asymptomatic clodronate-treated animals^6^. However, the immunomodulatory properties of circulating chromatin indicate that caution should be exercised when Clo-L or any cell death-promoting agent is employed as cell death products may alter immune cell production.

Another key question in sepsis has been the cellular source of systemic cytokines. Our data suggest that MZ macrophages are important contributors of cytokines found in the circulation during systemic infection. The signals required to trigger various cytokines differ. Extracellular chromatin is sufficient to induce IL-6 and IL-1β^10^. However, the induction of G-CSF requires the simultaneous presence of histones and fungal signals that are halmarks of an uncontrolled infection with a cytotoxic impact. During homoeostasis, splenic macrophages regulate granulopoiesis by monitoring the presence of apoptotic circulating neutrophils^50^. We now show that macrophage-mediated microbe capture also links the detection of systemic microbes and DAMPs to the regulation of neutrophil populations.

The selective loss of mature neutrophils has also been demonstrated in murine bacterial sepsis models and is linked to the receptor Triggering Receptor Expressed On Myeloid Cells Like 4 (TREML4)^22^. However, the upstream cell death inducing signals and the mechanistic basis for the selectivity between mature and immature cells remained unclear. Histones and G-CSF regulate this process by selectively targeting mature neutrophils and may be relevant in other conditions, given that alterations in the age of neutrophils influences neutrophil function^51–53^. The ability of G-CSF to shorten the lifespan of mature neutrophils uncovers a new mechanism for the regulation neutrophil populations through the modulation of lifespan in addition to granulopoiesis and BM egress. In our sepsis patient survival analysis, G-CSF and neutrophil lifespan shortening activity correlated with mortality whereas plasma histones or DNA did not, despite their correlation with plasma S100A8/9 concentrations. These findings reflect the intricacies of the upstream regulation of G-CSF expression the potential for G-CSF to play both beneficial and detrimental roles during systemic infection that may depend on its concentration and the stage of infection. Evidently, the further downstream one assesses the pathway the more accurate becomes the survival prognosis. The combination of G-CSF and high neutrophil lifespan shortening activity proved even more informative, with the latter being able to predict survival in patients with medium levels of plasma G-CSF. These readouts may be helpful in directing new therapies for sepsis patients. Furthermore, S100A8/9 proteins induce pathogenic neutrophil subsets in severe COVID-19 infection, suggesting that the release of these alarmins could further impact neutrophil populations and disease severity^24^

The loss of mature neutrophils should expand the size of hematopoietic niches in the BM^50,54^. However, our analysis indicated loss of progenitors which may contribute to decreases in immature cells and may be linked to ROS deficiency, given that ROS production is required for the expansion of progenitors during emergency granulopoiesis^55^. Moreover, the elevated G-CSF levels may increase mature neutrophil egress in WT mice but cannot alone explain the loss of mature cells from the BM since mature neutrophils were also substantially absent in the periphery.

The transcriptional profile of Ly6G^low^ neutrophils in symptomatic WT mice suggests that they are immature cells with additional infection-associated transcriptional changes denoted by a distinct signature compared to Ly6G^low^ neutrophils in asymptomatic TCRα-deficient mice. G-CSF and Clo-L administration recapitulates the loss of mature to immature neutrophil balance, but it is evident that infection promotes additional changes in mature and immature neutrophils. Both immature populations in infected WT mice and sterile Clo-L and G-CSF treated mice upregulate arginase 1 (*Arg1*) expression unlike immature neutrophils in infected TCRα-deficient mice but differ by other surface markers such as CD14 that is only upregulated in all cells from symptomatic WT infected animals. The basis for the different sensitivity of mature and immature neutrophils to lifespan-shortening is unclear. Several transcriptional adaptations in the Ly6G^low^ neutrophil population of symptomatic mice may be important for the resistant of these cells to lifespan shortening. The downregulation of *Csf3r* transcript appears to be the result of a negative feedback response to hyper-activation of G-CSFR since it observed in both mature and immature neutrophils from infected symptomatic mice but not cells from naïve or infected asymptomatic TCRα-deficient mice. However, these changes were not reflected at the protein level. Both mature and immature neutrophils from infected symptomatic mice maintained their surface protein expression of G-CSFR but only immature cells remained resistant to the lifespan reduction, suggesting that G-CSFR has slow turn-over rates and other downstream mechanisms account for the resistance to G-CSF-mediated lifespan shortening observed in immature cells. The downregulation of *Gsdmd* and *Dfna5* transcripts and the upregulation of the anti-apoptotic factor *Bcl2* may render immature Ly6G^low^ neutrophils more resistant to cell death. Moreover, the downregulation of *Clec2d* may provide another mechanism that reduces Ly6G^low^ neutrophil exposure to histones given that this receptor mediates the uptake of extracellular chromatin^56^.

In addition, several changes in gene expression may reflect poor antimicrobial function of Ly6G^low^ neutrophils in symptomatic mice. Several important genes for antimicrobial defence exhibit relatively low expression in these cells, including *Clec7a* (Dectin-1) which is critical for fungal recognition and IL-1β which orchestrates neutrophil recruitment and swarming^57,58^. Notably, Ly6G^low^ neutrophils exhibited lower superoxide production despite the expression of all major NADPH oxidase genes. Ly6G^low^ neutrophils exhibited a higher expression of *Mpo* transcript and protein compared to Ly6G^high^ neutrophils allowing these cells to be distinguished from mature neutrophils in the BM by microscopy. Lower ROS production also renders neutrophils more proinflammatory in fungal and bacterial infections which may contribute to sepsis pathology^58^. However, the abundance of NETs in the kidneys of symptomatic mice, suggests that NETosis is not disrupted and may be driven either by the remaining minor mature neutrophil population or immature neutrophils with a sufficient ROS burst. On the contrary, anti-SIGNR1 treatment reduced NETosis possibly by direct control of fungi via ROS, which may further limit pathology.

Immune and neutrophil dysfunction in sepsis patient survivors can persist after recovery. The mechanism that we identify here acts predominately via the active elimination of mature neutrophils, rather than by reprogramming myelopoiesis. This mechanism could be relevant in the chronic phase of recovery if it were driven by the persistence of cell-free chromatin and G-CSF in the circulation and splenic macrophage reprogramming.

The pathogenic alteration of neutrophil populations by G-CSF and circulating histones may be relevant in other chronic conditions such as cancer. Tumour-derived G-CSF is required but not sufficient to upregulate a minor population of tumour-induced pro-metastatic low-density granulocytes^39,59,60^. Tumour-derived DAMPs and G-CSF may drive pro-tumorigenic neutrophil populations and may provide further mechanistic understanding for the therapeutic potential of DNase treatment^61–64^. Consistently, in our experiments, Ly6G^low^ neutrophils from infected symptomatic mice have increased *Arg1* expression which inhibits T cell proliferation and promotes tumour growth. Moreover, neutrophils from healthy human donors upregulated PD-L1 surface protein levels when incubated with sepsis plasma, G-CSF or histones, indicating that these conditions could enhance their pro-tumorigenic capacity. A sub-population of neutrophils with upregulated PD-L1 has also been described in COVID-19 patients and thus this mechanism may drive altered neutrophil populations in severe COVID-19 patients, where circulating chromatin and hyperinflammation have also been reported^65,66^.

Overall, our study demonstrates the close connection between immune dysfunction in T cells and neutrophils that can be targeted by interfering with microbial sequestration in the spleen or by eliminating circulating chromatin. The presence of G-CSF and circulating histones in many conditions suggests that they may be critical drivers of immune dysfunction that could be targeted therapeutically.

## Methods

### Informed consent and ethics for human samples

For in vitro neutrophil experiments, peripheral blood was isolated from consenting healthy adult volunteers, according to approved protocols of the ethics board of the Francis Crick Institute and the Human Tissue act. Sepsis patient samples were provided by the Hannover Medical School approved by the ethics committee under the study protocol (trial registration: NCT04231994, protocol No. 2786-2015). Written informed consent was obtained from participants or authorized representatives. The study was performed in accordance with the ethical standards laid down in the 1964 Declaration of Helsinki and its later amendments. No funding specific to this project was received.

### Animals

All mice were bred and maintained in a pathogen free, 12 h light-dark cycle environment. All experiments were conducted with age-matched and cage-controlled, 8–12-week-old female WT C57BL/6 J, MPO−/− (B6.129X1-*Mpo*^*tm1Lus*/J^), TCRα−/− (B6.129-Tcra^*tm1Phi*^), Rag2^−/−^ (B6.129S6-Rag2^tm1Fwa^ N12), G-CSF−/−(Csf3^tm1Ard^) and FVB/NJ mice, according to local guidelines and UK Home Office regulations under the Animals Scientific Procedures Act 1986 (ASPA). Mice were monitored twice a day, in the early morning and late afternoon. Mice with temperatures below 32 °C exhibited signs of severe systemic inflammation as they were hunched, immobile, had a scruffy coat and would not survive longer than a few hours and were sacrificed as these had reached the established humane endpoints.

### Neutrophil isolation from peripheral human blood

Peripheral venous blood was collected into EDTA tubes, layered on Histopaque-1119 (Sigma-Aldrich) and centrifuged for 20 min at 800 × *g*. The plasma, PBMC and neutrophil layers were collected and neutrophils were washed in Hyclone Hank’s Balanced Salt Solution (HBSS) without calcium, magnesium or phenol red (GE Healthcare) supplemented with 10 mM HEPES (Invitrogen) 0.1% plasma and further fractionated on a discontinuous Percoll (GE Healthcare) gradient consisting of layers with densities of 1105 g/ml (85%), 1100 g/ml (80%), 1093 g/ml (75%), 1087 g/ml (70%), and 1081 g/ml (65%) by centrifugation for 20 min at 800x g. Neutrophil enriched layers were collected and washed.

### In vitro stimulation of human neutrophils for FACs analysis

2 × 10^5^ neutrophils from human peripheral blood were seeded in a U-bottom 96-well plate in HyClone HBSS + Ca, + Mg, − Phenol red (GE Healthcare) supplemented with 10 mM HEPES (Invitrogen) and 3% plasma from healthy or septic donors. In vitro stimulation was performed with 500 nM of human recombinant Histone 3 (Cayman chemical) and 5 ng/ml recombinant human G-CSF (BioLegend). The cells were then incubated for 16 h at 37 °C and 5% CO_2_. Cells were then washed, stained and fixed for flow cytometry analysis according to the steps described below.

### Time-lapse imaging and half-life quantification

5 × 10^5^ neutrophils from human peripheral blood were seeded in a black 96-well plate in HyClone HBSS + Ca, + Mg, - Phenol red (GE Healthcare) supplemented with 10 mM HEPES (Invitrogen) and 3% plasma from healthy or septic donors. For mouse experiments, 5 × 10^5^ flow cytometry sorted BM neutrophils were incubated with Roswell Park Memorial Institute 1640 medium (RPMI; Thermo Fisher Scientific) supplemented with 10 mM HEPES (Invitrogen) and 3% fetal calf serum (FCS; Sigma). To distinguish between live and dead cells, we stained with 5uM Hoechst (membrane permeable; Thermo Scientific) and 5uM Sytox-green (membrane impermeable; Invitrogen). The cells were imaged on a long-term time-lapse wide-field Nikon system in a temperature (37 °C) and CO_2_ (5%) regulation chamber. Four fields of view were acquired per well every 30 mins for 32 hrs using a 40x objective.

Quantification of number of Sytox^+^ cells over total cells were performed using intensity-based thresholding masks in Fiji and particles between 20–1200 µm were quantified. % Sytox positive/total cells were fit with a sigmoidal function using Prism 9 to obtain the neutrophil half-lives. In vitro stimulation was performed with 100 nM, 500 nM and 1uM of human recombinant Histone 3 (Cayman chemical) and 1 ng/ml, 5 ng/ml or 50 ng/ml recombinant human G-CSF (BioLegend) or mouse G-CSF (BioLegend). The blocking of H3 and G-CSF was performed by preincubating the different 3% plasma with 0.5 µg/ml of anti-hH3, 1 µg/ml and of anti-human G-CSF or 1.5 µg/ml of anti-IgG at 37 °C for 30 mins before adding to the neutrophils.

### Murine candidiasis and *S. aureus* sepsis model

Wild-type *Candida albicans* (*C. albicans*, clinical isolate SC5314) was cultured overnight shaking at 37 °C and sub-cultured to an optical density (OD600) of 0.4–0.8 for 4 h in yeast extract peptone dextrose (YEPD; Sigma) medium. Subcultures were examined for lack of hyphae, washed and resuspended in sterile phosphate-buffered saline (PBS) immediately prior to infection. Mice were injected intravenously with either 1 × 10^3^, 1 × 10^4^, 1 × 10^5^ or 5 × 10^5 ^*C. albicans* yeast particles per mouse. *Staphylococcus aureus* strain 8325-4 was cultured overnight in LB medium at 37 °C on a shaker. A 1:100 subculture was made to an OD600 = 0.08–0.1 and the bacteria were grown for 4 h. 1 × 10^6^ CFUs were injected intravenously in the tail vein.

The weight and rectal temperature of the mice were recorded prior to infection and daily over the course of infection to track health status. A body temperature between 20–32 °C, a weight loss superior to 80% of initial weight accompanied by slow movement and non-responsiveness were considered collectively as septic shock and the humane endpoint for the mice. The mice were culled via cervical dislocation or by lethal dose of pentobarbital (600 mg/kg) with mepivacaine hydrochloride (20 mg/ml).

### Neutrophil and macrophage depletion in vivo

Neutrophil depletion was achieved with intraperitoneal injection of 150 μg anti-Ly6G Ab (BioXCell) or IgG isotype control (BioXCell) at day −1 and day 0 (day of infection). Macrophage depletion was performed with intravenous administration of 1 mg Clodronate liposomes (Clo-L) or 1 mg PBS liposomes (PBS-L) as control (Liposoma) at 1 day prior to infection.

### Treatment with blocking and neutralizing antibodies

SIGNR1 blocking was performed with intraperitoneal injections of 100 μg anti-SIGNR1 Ab (BioXCell) or hamster IgG isotype control (BioXCell) 1 day before infection followed by intraperitoneal injections (100 μg/mouse) every other day. G-CSF neutralization was achieved with intraperitoneal injections of 100 μg a-G-CSF (eBioscience) or IgG2a isotype control (eBioscience) started 1–2 days post-infection and performed every day until termination of the experiment. Histone neutralisation experiments were performed via intraperitoneal injections with dialysed and combined a-Histone 3 and a-Histone 4 antibodies (Merck Millipore) or control polyclonal rabbit IgG (BioXCell), starting on D-1 (200 μg/mouse) and daily afterwards (200 μg H3 and 100 μg H4).

### Treatment with recombinant proteins and enzymes

Recombinant mouse GCSF (BioLegend) was injected intraperitoneally (2.5 μg/mouse) 1 day prior (D-1) or on the day of infection (D0) as indicated. For degradation of circulating DNA in vivo, mice were treated with deoxyribonuclease I (DNase I) from bovine pancreas (Sigma, 2000 units/mouse) or PBS vehicle control, intraperitoneally 1 day prior to infection and daily until completion of the experiment.

### Adoptive transfer experiments

T cells were isolated from naïve spleens with the EasySep T cell isolation kit (STEMCELL Technologies). For each mouse 4 × 10^6^ naïve T cells were injected intravenously via the tail, 2 days prior to infection.

### Induction of peripheral Ly6G^low^ neutrophils in naïve mice

Mice were injected intravenously with 1 mg of Clo-L or PBS-L (Liposoma) and the subsequent day intraperitoneally with 2.5 μg rG-CSF (BioLegend) or vehicle (PBS). Analysis of neutrophil populations was performed 2 days after rG-CSF injection, according to the methods described in flow cytometric analysis.

### *Candida albicans* preparation for cell culture experiments

Wild-type *C. albicans* was cultured overnight shaking at 37 °C in YEPD and then the following day sub-cultured to an optical density (A_600_) of 0.4–0.8 in YEPD (for yeast) or Roswell Park Memorial Institute 1640 medium (RPMI; Thermo Fisher Scientific) (for hyphae formation) for 4 h. Cultures were checked for lack of hyphae (for yeast preparations) or for complete germination into hyphae and were then washed in PBS and counted. For hyphal growth assay live fungi were used and for in vitro stimulation of BMDMs, fungi were inactivated for 15 min at 98 °C.

### Hyphal growth assay

BM neutrophils were collected from the femur and tibia of naïve WT C57BL/6 J and MPO-deficient mice using the EasySep mouse neutrophil isolation kit (STEMCELL Technologies). 2 × 10^5^ neutrophils were plated in Hank’s Balanced Salt Solution (HBSS) without calcium, magnesium or phenol red (Fisher Scientific) supplemented with 3% murine plasma and 10 mM HEPES (Sigma) and left to settle for 45 min in the incubator at 37 °C and 5% CO_2_. Subsequently, 5 × 10^4^ hyphal units were added and the samples were imaged overnight with time-lapse microscopy on a Leica DMIRB microscope (20x objective) fitted with an incubation chamber at 37 °C and 5% CO_2_. Image analysis and quantification of hyphal length was performed with Fiji/ImageJ version 2.0.0 software.

### In vitro BMDM stimulation

BM was collected from femurs and tibias of wild-type mice and cultured in Iscove’s Modified Dulbecco’s Medium (IMDM) + 30% L929 conditioned media + 10% FBS + 1% Penicillin/Streptomycin (P/S). After 6 days of culture, BM-derived macrophages (BMDMs) were detached with PBS + 2 mM EDTA, and 3 × 10^5^ cells per well were seeded in 24-well plates in IMDM + 10%FBS + 1%P/S. The next day, cells were washed to fresh IMDM + 10%FBS + 1%P/S media and stimulated overnight with increasing concentrations of human histone H3 (Cayman) and/or heat inactivated *C. albicans* hyphae or yeast at multiplicity of infection (MOI) of 2. Culture supernatants of stimulated BMDMs were collected and spun at 300*x*g for 10 min at 4 °C. Supernatants were analysed for cytokines as described below.

### Tissue fungal burden

Organs were homogenized with a tissue homogenizer in PBS and plated in various dilutions on Sabouraud dextrose agar plates with 100 μg/ml streptomycin to prevent growth of bacteria. The plates were incubated at 37 °C for 12–18 h and colony forming units (CFUs) were counted.

### Cytokines

Organs were homogenized with a tissue homogenizer in lysis buffer: PBS containing 1x proteinase inhibitors (Sigma-Aldrich) and 0.1% Nonidet P-40 (NP40; Sigma). Cytokine and chemokine analysis from organ lysates and plasma were performed with the mouse 36-plex ProcartaPlex cytokine/chemokine array kit (ThermoFisher Scientific), G-CSF mouse or human ProcartaPlex Simplex Kit (ThermoFisher Scientific), G-CSF ELISA kit (R&D Systems), IL-6 mouse ELISA kit (ThermoFisher Scientific) and IL-1β mouse ELISA kit (ThermoFisher Scientific), following manufacturer’s instructions. All ProcartaPlex samples were analysed using the Luminex Bio-Plex 200 system. ELISA plate results were measured with light absorbance at 450 nm using a plate reader (Fluostar Omega, BMG labtech).

### Histology and Immunofluorescence imaging

Freshly extracted organs were embedded in optimal cutting temperature (OCT) compound cryo-embedding media (VWR Chemicals BDH) and flash-frozen in a dry ice/100%ethanol slurry. Frozen sections (8 μm thickness) were dried, fixed in 4% paraformaldehyde (PFA; Sigma) and permeabilized with 0.5% Triton X-100 in PBS. Non-specific binding was blocked with 2% BSA (Sigma) and 2% donkey serum (Sigma) in PBS. Samples were then stained with 4′,6-diamidino-2-phenylindole dihydrochloride (DAPI; Life Technologies) and the following primary antibodies: anti-B220 (BioLegend), anti-Candida (Acris), anti-CD3 (BioLegend), anti-CD4 (BioLegend), anti-CD169 (BioLegend), anti-F4/80 (BioLegend), anti-Ly6G (BioLegend), anti-MARCO (BMA Biomedicals), anti-MPO (R&D Systems), anti-SIGNR1 (eBioscience), anti-TCR-β (eBioscience), anti-cleaved-caspase 3 (Cell Signalling), anti-cleaved-caspase 8 (Cell Signalling) and anti-*S.aureus* (Thermofisher). When required, fluorescently labelled secondary antibodies were used: donkey anti-rabbit IgG (Invitrogen) and goat anti-rat IgG (Invitrogen).

For terminal deoxynucleotidyl transferase dUTP nick end labelling (TUNEL), the Click-iT TUNEL Alexa Fluor 594 Imaging Assay kit (Invitrogen) was used. Cryosections(8 μm) were dried, fixed in 4% PFA and permeabilized with 0.5% Triton X-100 in PBS. The continuing steps were performed as stated by the manufacturer and afterwards the slides were stained with primary and secondary antibodies as indicated previously.

RNAscope in situ hybridization assay was performed on frozen sections (8 μm) using RNAscope 2.5 LS probes for G-CSF and IL-6 (Mm-CSF3-C2, cat. 400918, Mm-IL6 cat. 315898-C1, ACD/Bio-Techne) using the automated Assay for Leica Systems and according to the manufacturer’s instructions. Subsequently, the slides were stained with primary and secondary antibodies following the methods described above. Pan-caspase labelling was performed using the Poly-Caspase Assay Kit (MyBioSource) according to the instructions provided by the manufacturer.

All stained tissue sections were mounted in ProLong Gold (Molecular Probes). Images were taken using the Leica TCS SP5 inverted confocal microscope (20x, 40x, 63x original magnification) and analysis was performed using Fiji/ImageJ version 2.0.0 software.

A full antibody list is included in Supplementary Table 1.

### Tissue dissociation and preparation of single cell suspension

Kidneys were chopped up and incubated in a digestion medium containing 0.2 mg/ml Liberase TL (Roche) and 0.1 mg/ml DNase I (Roche) for 20 min while shaking at 37 °C. The digested kidney tissue was filtered and centrifuged in a Percoll (GE Healthcare) density gradient (40%/70%). Spleens were gently meshed in FACs buffer (PBS containing 3% FCS from Sigma) using a 40 μM cell strainer to prepare single cell suspensions. Whole blood was centrifuged in Histopaque-1119 (Sigma) in order to isolate peripheral blood leukocytes and separate plasma. ACK (ammonium-chloride-potassium, GIBCO) lysing buffer was used in all tissue samples to eliminate remaining erythrocytes.

### Flow cytometry analysis and sorting

Single cell suspensions were stained for 25 mins at 4 °C with LIVE/DEAD^TM^ fixable dead cell stains (Thermofisher Scientific) or DAPI (Invitrogen) and the following fluorescent antibodies: anti-CD3, anti-CD19, anti-CD11b, anti-Ly6G, anti-Ly6C, anti-C5aR1, anti-CD66b, anti-CD101, anti-PD-L1 (all from BioLegend), anti-G-CSFR primary (R&D), anti-rat secondary (Invitrogen), anti-CD15, anti-CD16, anti-CD10 (all from BD Biosciences) and MPO (Hycult Biotech). Cells were fixed with 4% PFA for 25 min at 4 °C. For intracellular stainings, FoxP3 transcription factor staining buffer kit (eBiosciences) was used for fixation and permeabilization, following manufacturer’s instructions. The BD LSR Fortessa was used for acquisition of all samples and FlowJo software v10 for subsequent analysis.

For the Vβ T cell analysis, single cell suspensions were obtained as indicated above and the mouse Vβ TCR Screening Panel (BD Pharmingen) was used for staining, according to the instructions provided by the manufacturer.

For sorting, single cell suspensions were obtained as described prior and enriched with the EasySep mouse neutrophil isolation kit (STEMCELL Technologies). This semi-pure cell suspension was stained with fluorescently labelled antibodies via the methods described above and sorted with a 70uM nozzle (RNAseq and Giemsa stainings) or a 100uM nozzle (in vitro culture experiments) on the BD FACSAria cell sorter.

### Cytospin and Giemsa staining

For neutrophil characterization by nuclear morphology, 10^5^−10^6^ sorted neutrophils were cytospun on slides, fixed in methanol and stained with freshly prepared and filtered Giemsa staining buffer, containing Giemsa R (Ral Diagnostics). All slides were mounted in dibutylphthalate polystyrene xylene (DPX) and imaged using the ZEISS Axio Observer Z1.

### ROS assay

Mouse spleens were gently meshed using a 40 μM cell strainer and erythrocytes were lysed with ACK lysing buffer (GIBCO). Neutrophils were isolated via negative selection with the EasySep mouse neutrophil isolation kit (STEMCELL Technologies). A total of 1 × 10^5^ neutrophils were then incubated with the chemiluminescent probe luminol (Sigma) and horseradish peroxidase (HRP; Sigma) and stimulated with 100 nM PMA (Sigma). Reactive oxygen species (ROS) production was measured by HRP mediated oxidation of luminol, producing a chemiluminescent signal that is detected with an UV filter on a spectrophotometric microplate reader (Fluostar Omega, BMG labtech).

### Western blot analysis

Samples (protein extracts or plasma) were boiled in a sodium dodecyl sulphate (SDS) buffer containing dithiothreitol (DTT) and resolved by polyacrylamide gel electrophoresis (SDS-PAGE) on a Criterion TGX precast gel (Any-KD; Bio-Rad Laboratories). Proteins were then transferred to a polyvinylidene difluoride (PVDF) membrane (Bio-Rad Laboratories) via semi-dry transfer. Non-specific binding was blocked with 5% bovine serum albumin (BSA; Fisher Scientific) in tris-buffered saline with 0.1% Tween 20 (TBS-T). The membranes were blotted with anti-histone 3 (Milipore) and detected with HRP–conjugated goat anti-rabbit (Thermo Scientific). Finally, the membranes were incubated with enhanced chemiluminescent substrate (ECL; Thermo Fisher Scientific) and imaged with a chemiluminescence imaging systems (Bio-Rad) or developed after exposure onto an X-ray film (Kodak) and digitally scanned.

### DNA quantitation

DNA in plasma was quantified using the Quant-iTTM PicoGreen dsDNA assay kit (Thermo Fisher Scientific) following the instructions provided by the manufacturer. The fluorescent signal (excitation at 488 nm) was measured using a spectrophotometric microplate reader (Fluostar Omega, BMG labtech).

### Plasma sample preparation for proteomic analysis

24 (heparin-treated) plasma samples of WT naive, WT infected, TCRα −/− naive, TCRα −/− infected mice in equal replicates of six were used for proteomics analysis. The samples were randomised and plated in a 96-well plate (Eppendorf). The protocol used for protein/peptide extraction and proteomics analysis has been described in detail in Messner et al., 2020^67^. Briefly, 5 μL of plasma was denatured in 50 μl 8 M Urea (Honeywell Research Chemicals), 100 mM ammonium bicarbonate (ABC, Honeywell Research Chemicals) and reduced with 5 μL of 50 mM dithiothreitol (DTT, Sigma-Aldrich) at 30 °C for 1 h. Alkylation with 5 μL of 100 mM iodoacetamide (IAA, Sigma-Aldrich) followed, at 23 °C for 30 min in the dark. Then the samples were diluted with 340 μL of 100 mM ABC and 220 μL were added to trypsin solution (Promega) for protein digestion at a trypsin/protein ratio of 1/40 and incubated at 37 °C overnight (17 h). Quenching of digestion was done by the addition of 25 μL of 10% v/v formic acid (FA, Thermo Fisher Scientific). Rounds of solid phase extraction clean-up steps were performed with the use of C18 96-well plates (BioPureSPE Macro 96-Well, 100 mg PROTO C18, The Nest Group) as described previously in Messner et al. 2020^67^. Methanol (Fisher Chemicals), 50% v/v acetonitrile (ACN, Fisher Chemicals) or 0.1% v/v FA was used at each centrifugation step as required. After final elution, the collected peptide material was dried by a vacuum concentrator (Eppendorf Concentrator Plus) and redissolved in 50 μl 0.1% v/v FA, to be processed by liquid chromatography-mass spectrometry.

### Liquid chromatography-mass spectrometry

1 μg of protein digest (peptides) was injected and analysed on a nanoAcquity Liquid Chromatograph (Waters) coupled to a TripleTOF 6600 Mass Spectrometer (Sciex) at a flow-rate of 5 µl/min. Separation was achieved using a Waters HSS T3 column (150 mm × 300 µm, 1.8 µm particles) in 20-min non-linear gradients starting with 3% B up to 40% B (Buffer A: 0.1% v/v FA; Buffer B: ACN / 0.1% v/v FA). A data independent acquisition (DIA/SWATH) method was used, with MS1 scan from m/z 400 to m/z 1250 and 50 ms accumulation time followed by 40 MS2 scans of 35 ms accumulation time with variable precursor isolation width covering the mass range from m/z 400 to m/z 1250. Ion source gas 1 (nebulizer gas), ion source gas 2 (heater gas) and curtain gas were set to 30, 15 and 25 respectively. The source temperature was set to 450 °C and the ion spray voltage to 5500 V. Injections of samples took place in a random order.

The library was generated with “gas-phase fractionation” methodology out of pooled peptide digest of all mouse plasma samples with the use of a LC-MS/MS method as mentioned before. 3 μg of protein digest was separated with a 60-min linear gradient (3% B to 40% B). Injections were performed at the mass ranges of: 400–500 m/z, 495–600 m/z, 595–700 m/z, 695–800 m/z, 795–900 m/z, 895–1000 m/z, 995–1100 m/z, 1095–1250 m/z. The precursor selection windows were 2 m/z (1 m/z overlap). DIA-NN 1.7.10 proteomics analysis software was used for the library preparation with *Mus musculus* (mouse) UniProt (UniProt Consortium, 2019) isoform sequence database (UP000000589) to annotate the library^68,69^.

For protein quantification, raw data acquired were processed with DIA-NN 1.7.10 with the “robust LC (high precision)” mode with MS2, MS1 and scan window size set to 20ppm, 12ppm and 8 respectively.

### Preparation of samples for high-throughput RNA sequencing

Spleen leukocytes were extracted via methods described in the flow cytometry section. Following homogenization, samples were enriched using the EasySep mouse neutrophil isolation kit (STEMCELL Technologies) and subsequently stained with antibodies and isolated via flow cytometric-sorting. RNA extraction was performed with the RNeasy mini kit (Qiagen) and the libraries were prepared with the Nugen cDNA synthesis kit.

### Bioinformatics

Sequencing was performed on an Illumina HiSeq 4000 machine. The ‘Trim Galore!’ utility version 0.4.2 was used to remove sequencing adaptors and to quality trim individual reads with the q-parameter set to 20. Then sequencing reads were aligned to the mouse genome and transcriptome (Ensembl GRCm38 release-89) using RSEM version 1.3.0 in conjunction with the STAR aligner version 2.5.2^70,71^. Sequencing quality of individual samples was assessed using FASTQC version 0.11.5 and RNA-SeQC version 1.1.8^72^. Differential gene expression was determined using the R-bioconductor package DESeq2 version 1.24.0^73^. Gene set enrichment analysis (GSEA) was conducted as described in^74^.

### Statistical analysis

Single comparison statistical significance was assessed by an unpaired, two-tailed Mann–Whitney *t*-test. Log-Rank Mantel–Cox test was applied for analysis in survival experiments. Grouped data with multiple variables were analysed by two-way Anova. Statistical analysis was performed on GraphPad Prism software. *P*-values of <0.05 were considered significant: n.s. *p* ≥ 0.05, *p ≤ 0.05, **p ≤ 0.01, ***p ≤ 0.001.

### Reporting summary

Further information on research design is available in the Nature Research Reporting Summary linked to this article.

## Supplementary information

Supplementary Information

Reporting Summary

## Data Availability

The high-throughput neutrophil RNA sequencing expression profiles generated in this study are available at the NCBI Gene Expression Omnibus Database under the accession code GSE160301. The RNA sequencing data are available without restricted access. The raw RNA sequencing data are protected and are not available due to data privacy laws. The processed RNA sequencing data are available at NCBI Gene Expression Omnibus. The RNA sequencing data generated in this study are provided in the Supplementary Information/Source Data file. The mass spectrometry plasma proteomics data have been deposited to the ProteomeXchange Consortium via the PRIDE partner repository with the dataset identifier PXD034391. Source data are provided with this paper.

## Source data

Source Data
