# Supplementary material for: Microbe capture by splenic macrophages triggers sepsis via T cell-death-dependent neutrophil lifespan shortening
Source: Nat Commun. 2022 Aug 9;13:4658. doi: 10.1038/s41467-022-32320-1 (PMC9361272; doi:10.1038/s41467-022-32320-1)
Supplement: Supplementary file 1 — Supplementary Information [file 41467_2022_32320_MOESM1_ESM.pdf]

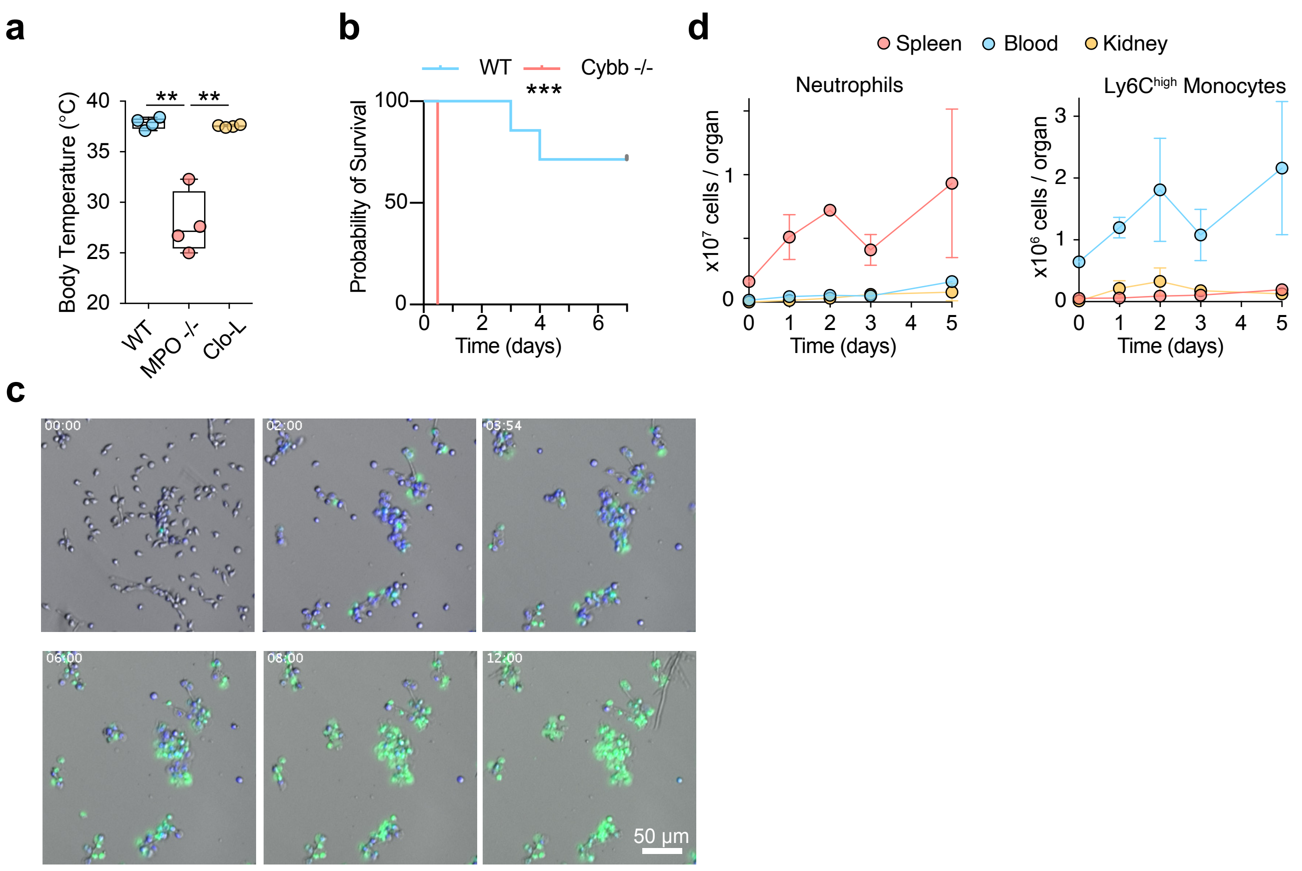

Supplementary Figure 1. Myeloperoxidase and the NADPH oxidase control fungal growth. a Body temperature of WT or MPO-deficient mice, or WT mice pre-treated with clodronate liposomes (Clo-L), infected intravenously with $1x10^5$ WT *C. albicans*, 12 hrs post-infection (n=4 mice per group). **b** Survival curves of WT and Cybb-deficient animals infected with $1x10^5$ WT *C. albicans* (WT n=7 and Cybb-/- n=6 mice). Representative of 2 independent experiments. **c** Timelapse microscopy images of *C. albicans* hyphae cultured with murine WT bone marrow-derived neutrophils in the presence of Hoechst (blue) and Sytox Green (green). Images were obtained every 2 min intervals for 12 hrs. Images from 0, 2, 4, 6, 8, 12 hrs are shown (n=4 hyphae particles examined over 12 hours). Representative data from 3 independent experiments. **d** Total number of neutrophils in the spleen (red), blood (blue) and kidneys (yellow) of WT mice infected intravenously with $1x10^5$ WT *C. albicans* at the indicated days post-infection, analysed by flow cytometry (n=3 mice / group and 2 experimental repeats). Statistical analysis by unpaired two-sided Mann-Whitney t-test (a) and Mantel-Cox log rank survival analysis (b) (* p<0.05, ** p<0.01, *** p<0.001, **** p<0.0001).

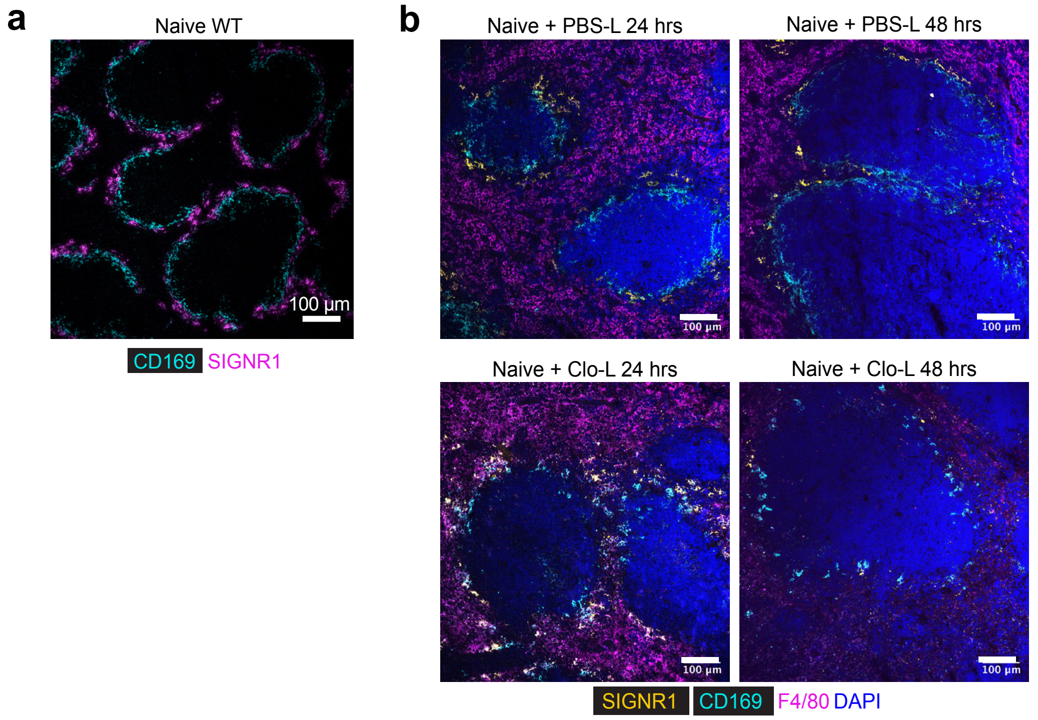

Supplementary Figure 2. Sensitivity of splenic macrophages to clodronate liposomes. Representative immunofluorescence confocal micrographs from: **a** the spleen of a naïve WT mouse, stained for CD169 and SIGNR1. Scale bars: 100 µm (n=3 naive mice analysed individually in 2 independent experiments) and **b** spleens from WT mice treated with PBS (PBS-L) or clodronate (Clo-L) liposomes for 24 hrs or 48 hrs, stained for CD169, SIGNR1, F4/80 and DAPI. Data from 2 independent experiments with 4 mice per group. Scale bars: 100 µm.

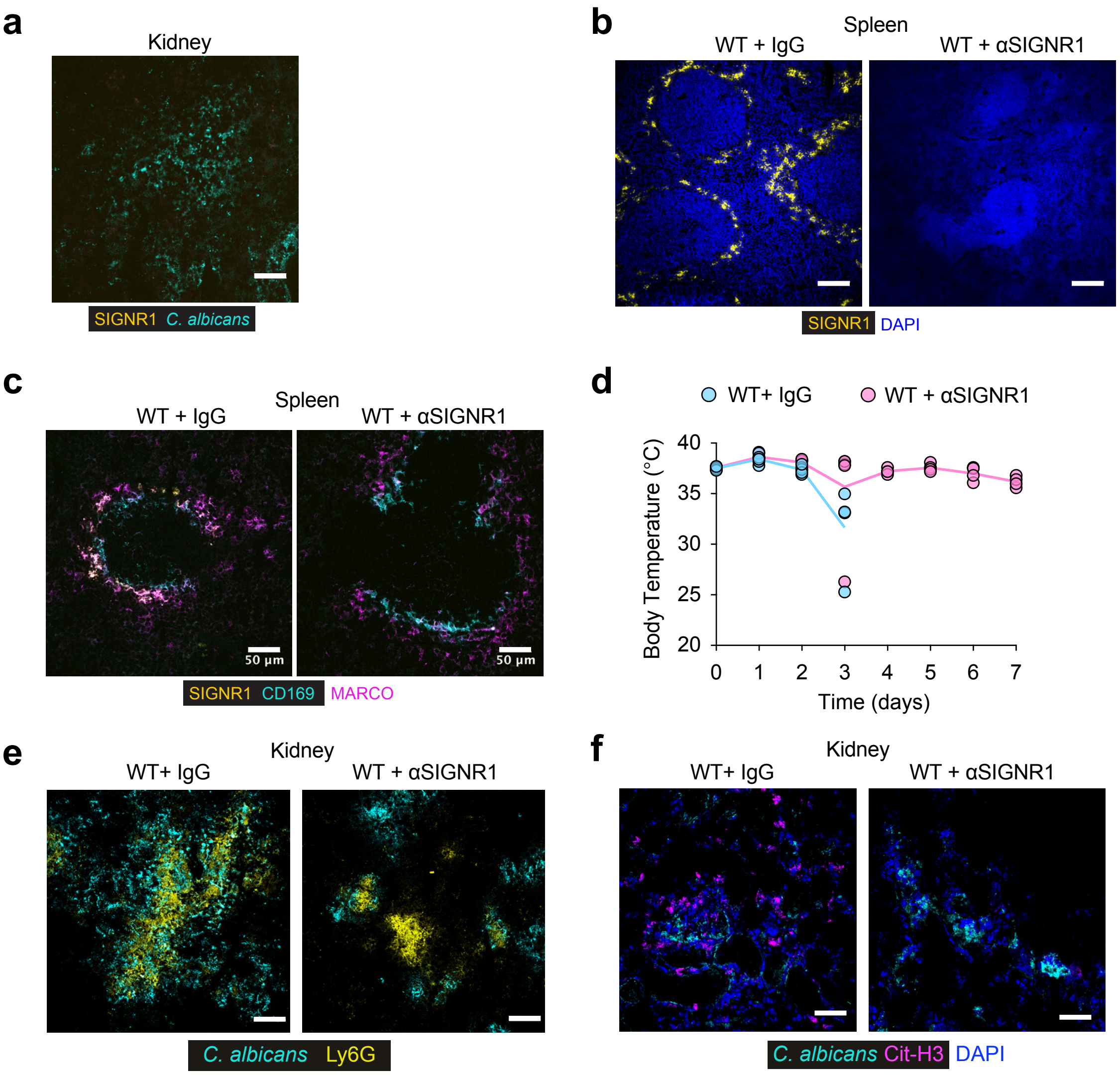

Supplementary Figure 3. Impact of anti-SIGNR1 treatment on immune populations and responses. **a** Representative immunofluorescence confocal micrograph from the kidneys of symptomatic WT mice infected intravenously with 5x10^5 WT *C. albicans* 3 days post-infection, stained for SIGNR1 and *C. albicans* (n=3 mice analysed individually and 2 experimental replicates). Scale bars: 50 µm. **b** Immunofluorescence confocal micrographs of spleens from naive WT mice treated with control or anti-SIGNR1 antibodies for 72 hrs and stained for DAPI and SIGNR1 (n=3 per group and 2 independent experiments). Scale bars: 50 µm. **c** Spleens from *C. albicans* infected mice, pre-treated with control or anti-SIGNR1 antibodies, harvested 72 hrs post infection and stained for SIGNR1, MARCO, CD169 and DAPI (n=4 mice analysed individually in 2 independent experiments). Scale bars: 50 µm. **d** Body temperatures of WT mice pre-treated with either control antibody (blue) or an anti-SIGNR1 antibody (magenta) infected intravenously with 5x10^5 WT *C. albicans*. **e** Micrographs of kidneys from WT mice pre-treated with either control or anti-SIGNR1 antibodies, 72 hrs after intravenous infection with 5x10^5 WT *C. albicans*, stained for *C. albicans* and Ly6G. 3 independent experiments with 3 mice analysed individually. Scale bars: 50 µm. **f** Micrographs of kidneys from (e) stained for cit-H3, *C. albicans* and DAPI. Scale bars: 50 µm.

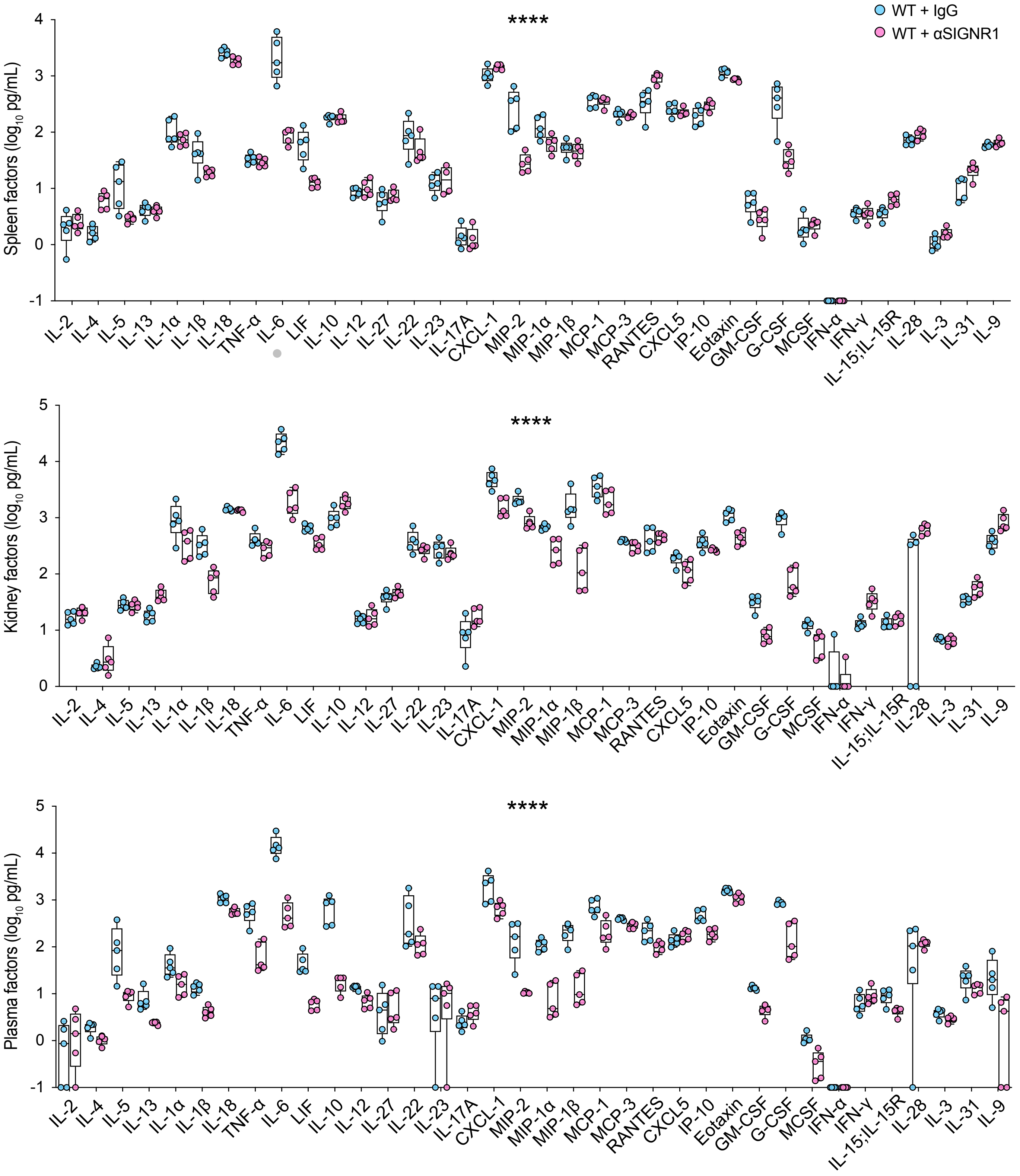

Supplementary Figure 4. SIGNR1 promotes systemic inflammation. Multiplex assay measurements of cytokines and chemokines in the spleens (top), kidneys (middle) and plasma (bottom) of WT mice pre-treated with either control (blue) or anti-SIGNR1 antibodies (magenta), infected intravenously with 5×10^5 WT *C. albicans,* 3 days post infection (n=5 mice per group). Statistical analysis by two-way Anova (ns >0.05, * p<0.05, ** p<0.01, *** p<0.001, **** p<0.0001).

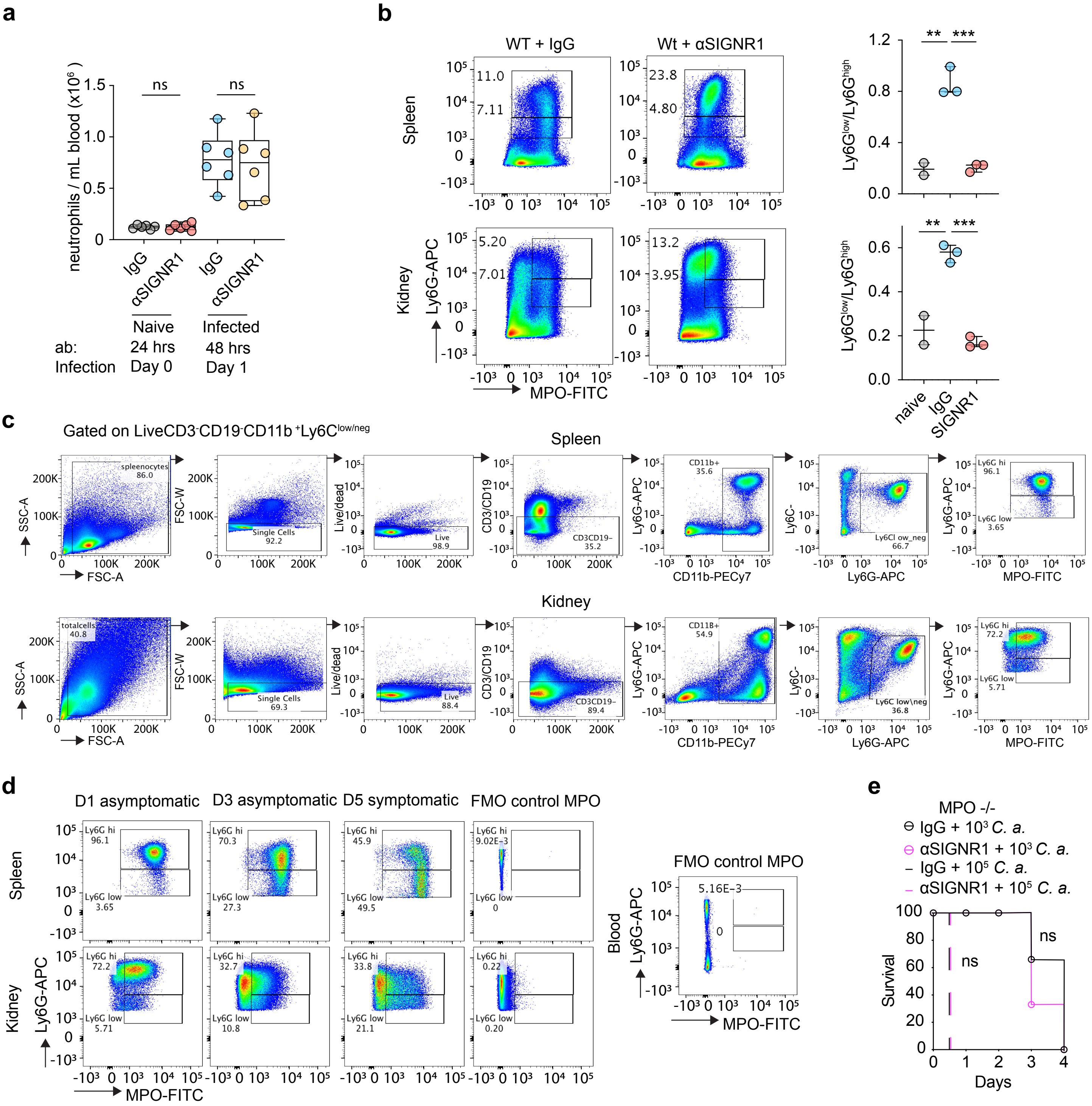

Supplementary Figure 5. SIGNR1 regulates peripheral neutrophil population maturity. a-b Analysis of samples from WT mice pre-treated with either control or anti-SIGNR1 antibodies and infected intravenously with 5x10^5 WT *C. albicans*. Representative results of 3 independent experiments. **a**. Total number of neutrophils per mL of blood of naïve mice on the day of infection (24 hrs after antibody administration) or infected mice 24 hrs post infection (48 hrs after antibody administration) (n=6 mice per group). **b** Flow cytometry graphs (left) and corresponding Ly6G^{low}/Ly6G^{high} ratios (right) of neutrophils in the spleen and kidneys of infected mice pre-treated with IgG control or anti-SIGNR1 antibodies, 72 hrs post infection and stained for intracellular MPO and surface Ly6G expression (naive n=2 and infected n=3 mice). **c** Gating strategy for flow cytometric analysis of neutrophils in the spleen and kidneys. **d** Flow cytometry of neutrophils in the spleen and kidneys of WT infected mice 24 hrs, 72 hrs and 5 days post-infection, in an experiment where WT mice started developing symptoms 5 days post-infection. **e** Survival of MPO-deficient mice pre-treated with either control or anti-SIGNR1 antibodies, infected intravenously with either 1x10^3 or 1x10^5 WT *C. albicans* (n=3 and n=5 mice per group). Statistical analysis by unpaired two-sided Mann-Whitney t-test for single comparison and two-way Log-rank Mantel-Cox test for survival analysis (ns >0.05, * p<0.05, ** p<0.01, *** p<0.001, **** p<0.0001).

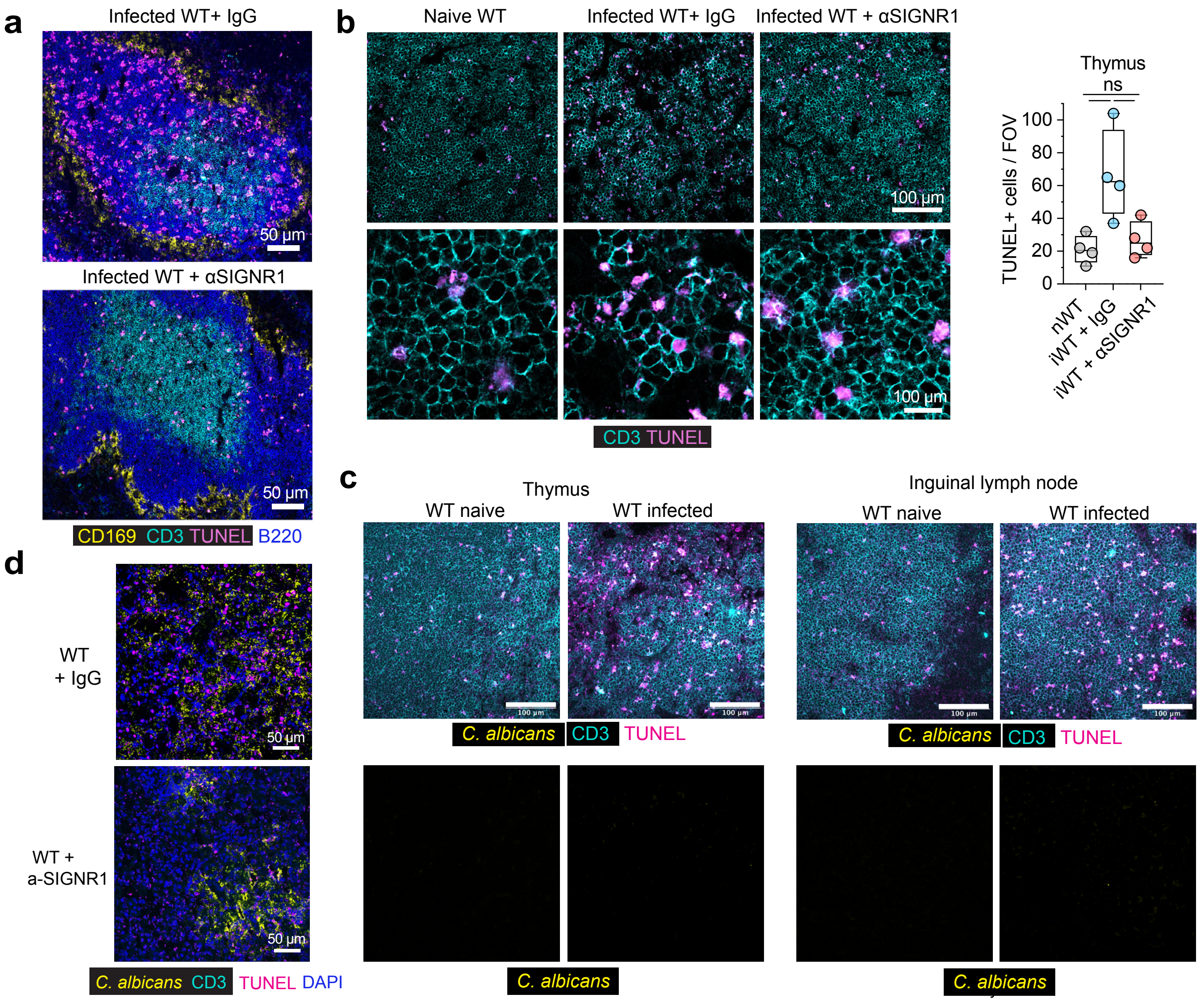

Supplementary Figure 6. SIGNR1 promotes cell death in the kidneys, thymus and lymph nodes. **a-d** Naïve WT mice or infected with 5x10^5 WT *C. albicans* and treated with control IgG or anti-SIGNR1 antibodies, 72 hrs post-infection **a** Immunofluorescence micrographs of spleens stained for appoptotic cells (TUNEL), CD3, CD169 and B220. Representative images of 3 animals per group and 3 independent experiments. Scale bars: 50 µm. **b** Immunofluorescence micrographs of the thymus stained for TUNEL and CD3. Quantitation of average TUNEL+ cells per FOV is depicted on the right (n=4 mice per group). Scale bars: 100 µm (upper row) and 25 µm (lower row). **c** Immunofluorescence confocal micrographs of the thymus and inguinal lymph nodes stained for TUNEL, CD3 and *C. albicans* (n=4 mice per group). Scale bar: 100 µm. **d** Immunofluorescence micrographs of kidneys stained for *C. albicans*, CD3, TUNEL, DAPI (n=3 mice per group). Data representative from 3 independent experiments. Scale bar: 50 µm. Scale bars: 50µm. Statistical analysis by two-sided unpaired Mann-Whitney t-test (* p<0.05, ** p<0.01, *** p<0.001, **** p<0.0001).

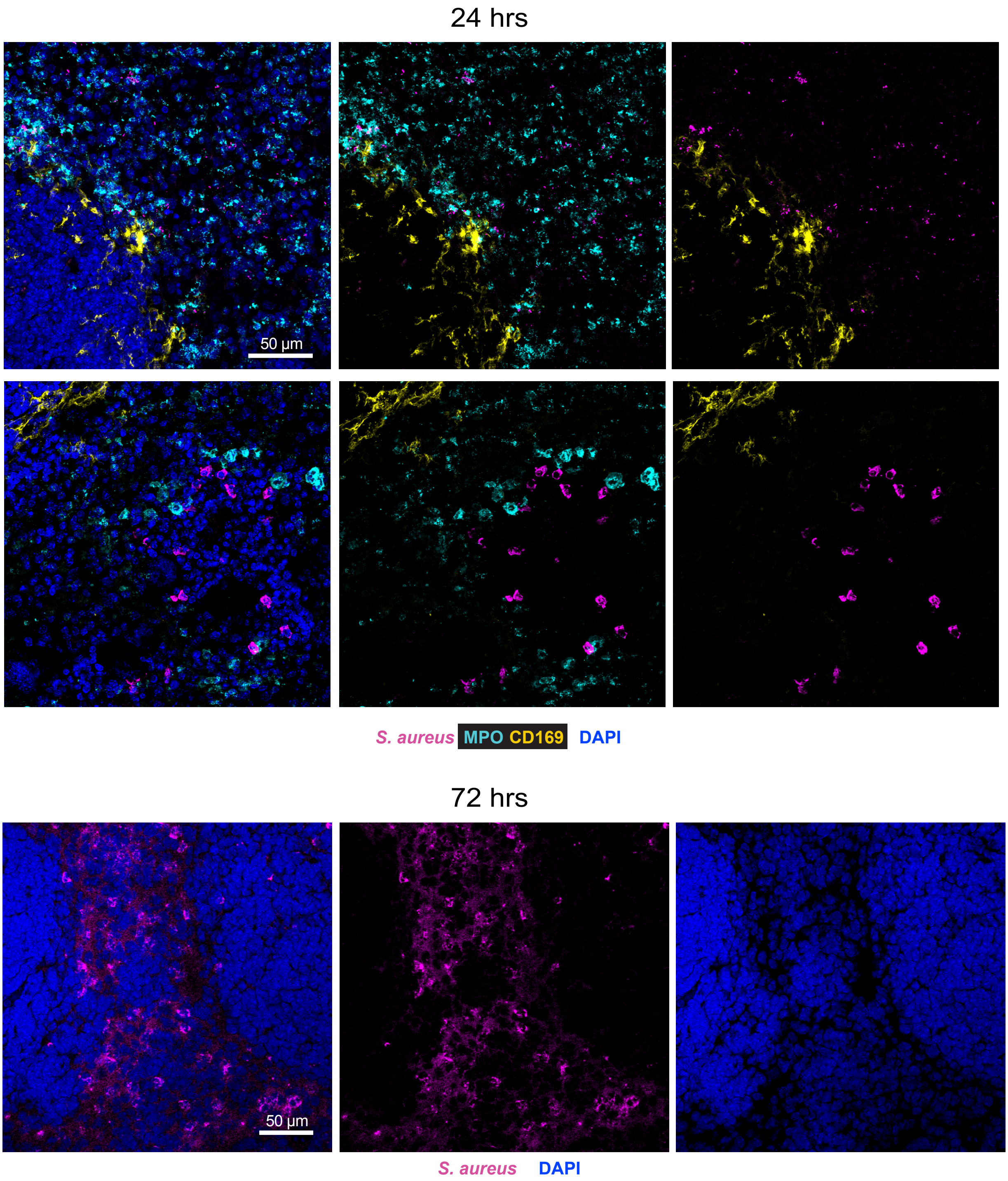

Supplementary Figure 7. *Staphylococcus aureus* colonization in the spleen. Representative confocal micrographs of spleens of WT mice infected with $1x10^6$ WT *S. aureus* 24 hrs (top) and 72 hrs (bottom) post-infection, stained for *S. aureus,* MPO, CD169 and DAPI (n=3 animals per group). Representative of 2 independent experiments. Scale bars, 50 µm.

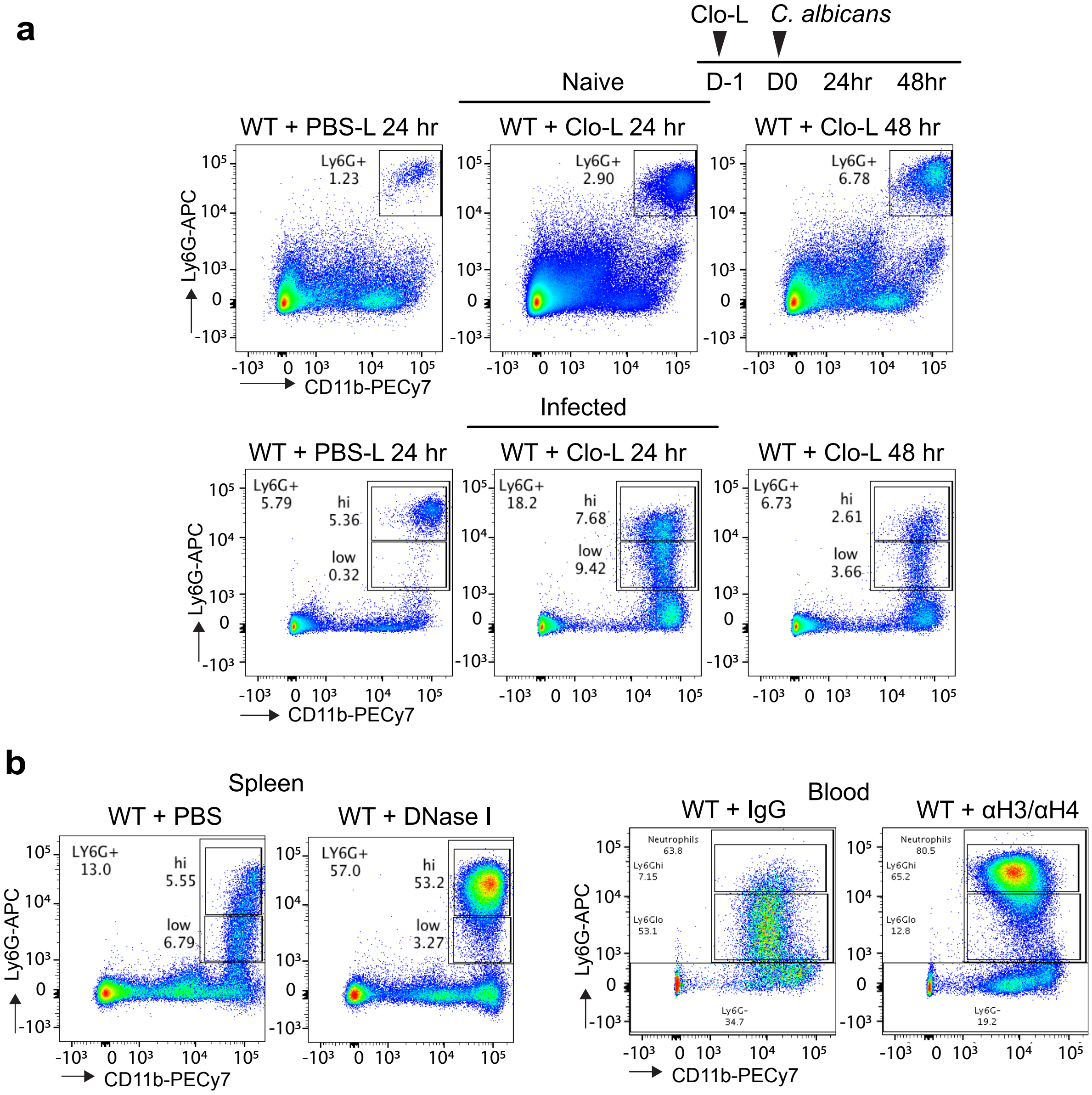

Supplementary Figure 8. Extracellular chromatin alters neutrophil populations. a Representative flow cytometry graphs depicting Ly6G^{low}/Ly6G^{high} ratios in the spleens of WT mice pre-treated with PBS-L or Clo-L, either naïve or infected with 1x10^5 WT *C. albicans*, 24 hrs and 48 hrs post-infection. **b** Representative flow cytometry graphs depicting Ly6G^{low}/Ly6G^{high} ratios in the spleen (left) and blood (right) of WT mice pre-treated with either DNase I, PBS, control IgG or anti-H3 and anti-H4 antibodies, infected intravenously with 5x10^5 WT *C. albicans*, 3 days post-infection.

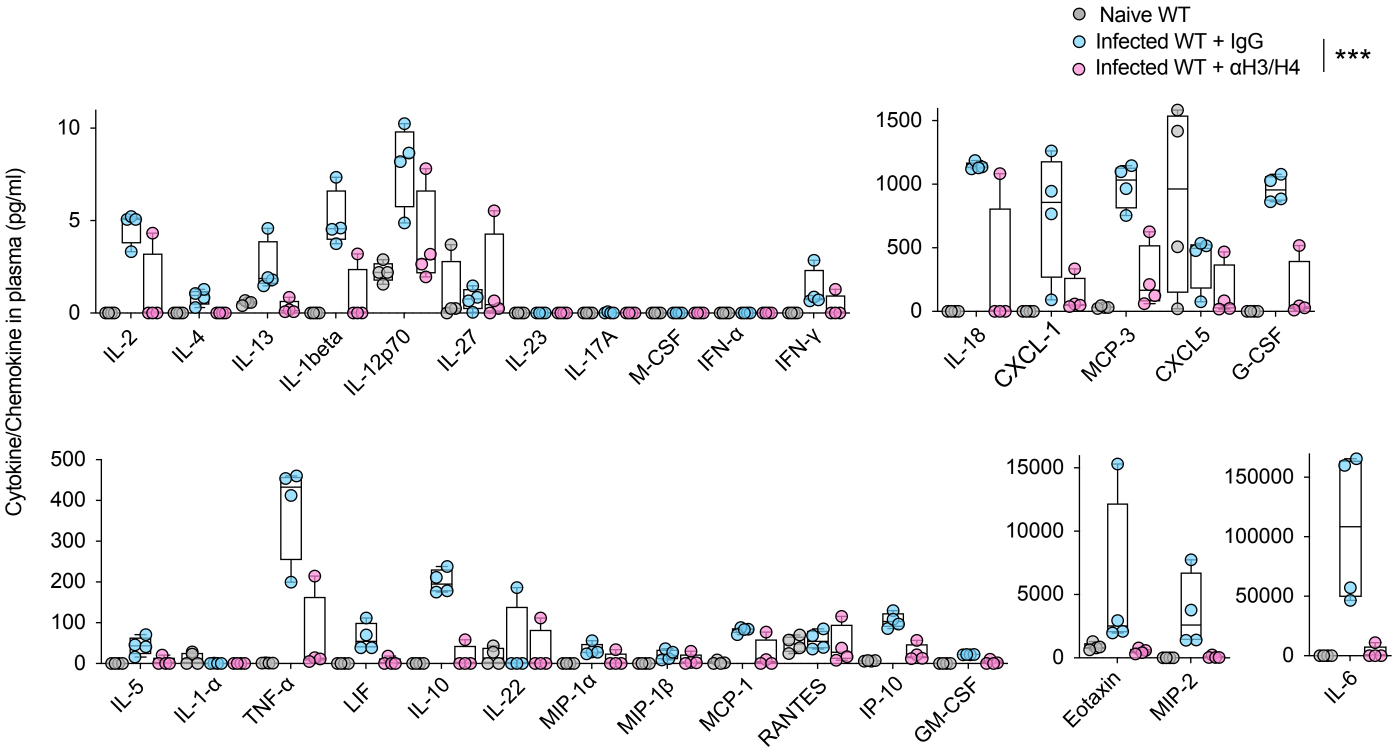

Supplementary Figure 9. Extracellular chromatin promotes systemic inflammation. Cytokines and chemokines measured by multiplex immunoassay in the plasma of WT mice either naive or pre-treated with IgG or anti-H3/anti-H4 antibodies and infected with 5×10^5 *C. albicans*, 72 hrs post-infection (n=4 mice per group). Statistical analysis by two-way Avova (* p<0.05, ** p<0.01, *** p<0.001, **** p<0.0001).

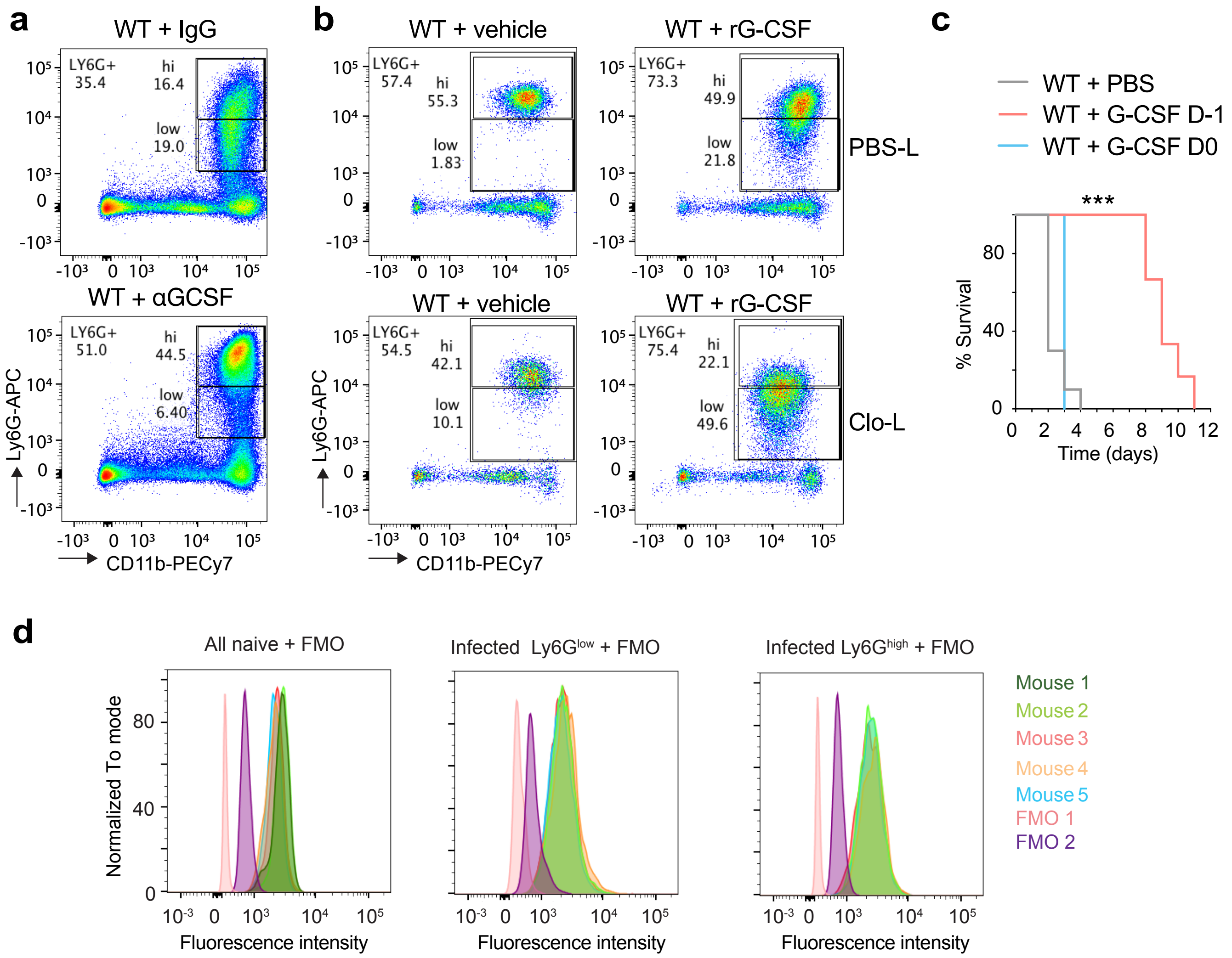

Supplementary Figure 10. G-CSF and cell death-derived chromatin alter neutrophil populations. **a** Representative flow cytometry graphs of Ly6G^{low}/Ly6G^{high} ratios in the spleens of WT mice infected intravenously with 5x10^{5} *C. albicans*, treated with control or anti-G-CSF antibody at 24 hrs and 48 hrs post-infection and analysed 72 hrs post-infection. **b** Representative flow cytometry graphs of Ly6G^{low}/Ly6G^{high} ratios of neutrophils in the spleens of naive mice pre-treated with PBS-L or Clo-L and administered PBS or recombinant G-CSF 24hrs later. Analysis performed 72 hrs after liposome administration. **c** Survival of WT mice pre-treated with PBS or recombinant G-CSF, administered 24 hrs prior to (D-1) or on the day of infection (D0) with 5x10^{5} WT *C. albicans*. **d** Flow cytometry analysis of G-CSFR surface protein expression in Ly6G^{high} neutrophils from naïve WT mice (left panel), or Ly6G^{low}(middle panel) and Ly6G^{high} (right panel) neutrophils from infected mice 3-4 days post-infection. Ly6G^{high} neutrophils of naïve mice stained in the absence of primary and secondary antibodies (FMO1) or with secondary antibody alone (FMO2). Statistical analysis by two-sided Log-rank (Mantel-Cox) test for survival analysis (* $p<0.05$, ** $p<0.01$, *** $p<0.001$, **** $p<0.0001$).

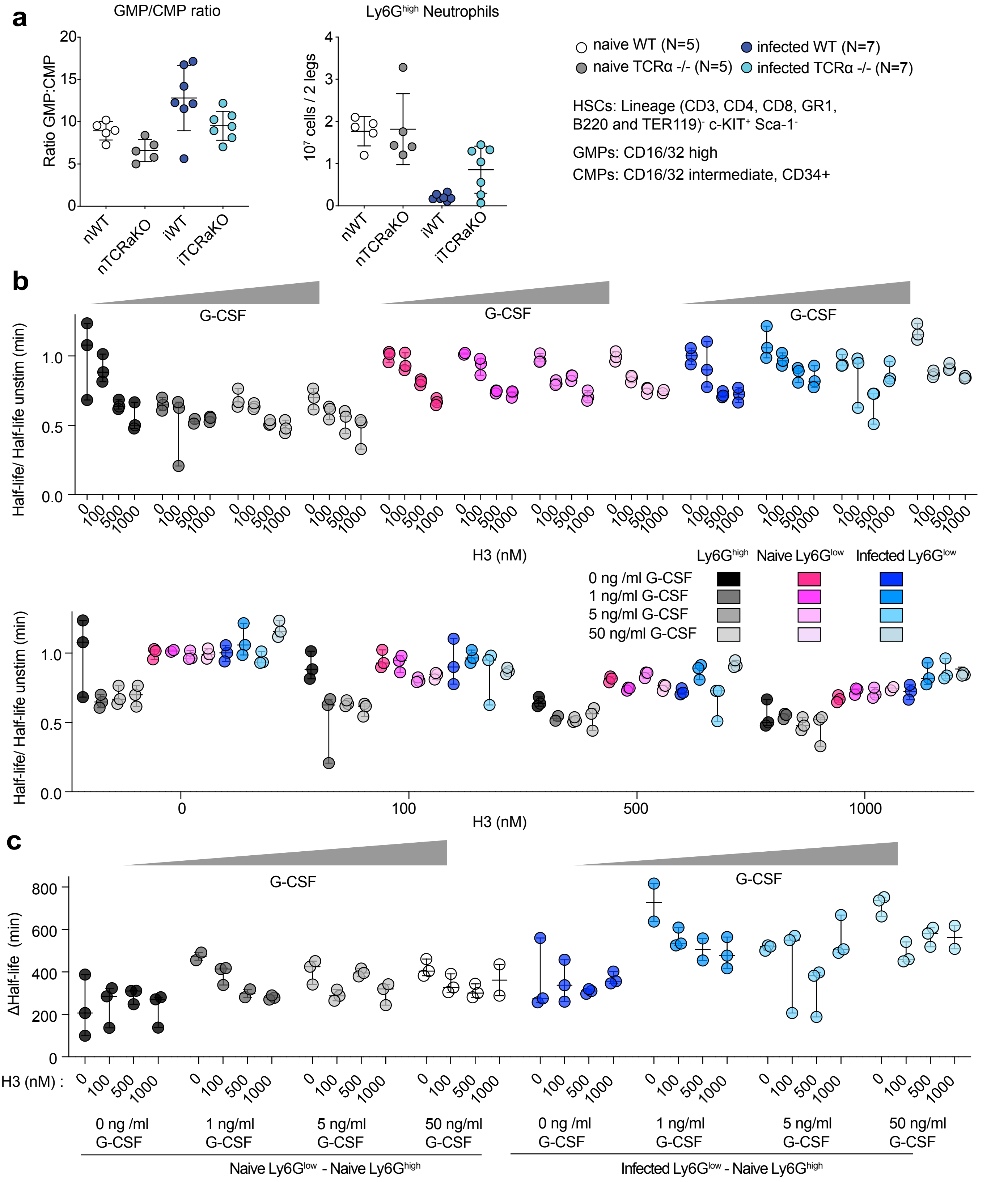

Supplementary Figure 11. Effect of G-CSF and histones on neutrophil progenitors and neutrophil lifespan. a Flow cytometry analysis of myelopoietic progenitors and mature Ly6G^{high} neutrophils in the bone marrows of naive WT and TCRα-deficient animals or infected intravenously with 5x10^{5} *C. albicans*. The left panel depicts the ratios of GMPs to CMPs and the right panel the corresponding total Ly6G^{high} neutrophils in the bone marrow (naive n=5 and infected n=7 mice per group). **b** Half-life ratio of Ly6G^{high} and Ly6G^{low} neutrophils from naive mice or Ly6G^{low} neutrophils from infected symptomatic mice supplemented with histone H3 or G-CSF at the indicated concentrations in the color scheme label. Average and SD from 3 FOV per condition. **c** Difference in the half-life between WT naive Ly6G^{high} and Ly6G^{low} neutrophils (black and grey shades) or WT naive Ly6G^{high} and Ly6G^{low} neutrophils from infected symptomatic mice (blue shades) alone or supplemented with histone H3 or G-CSF at the indicated concentrations. Average and SD from 3 FOV per condition. Data are representative of 3 independent experiments.

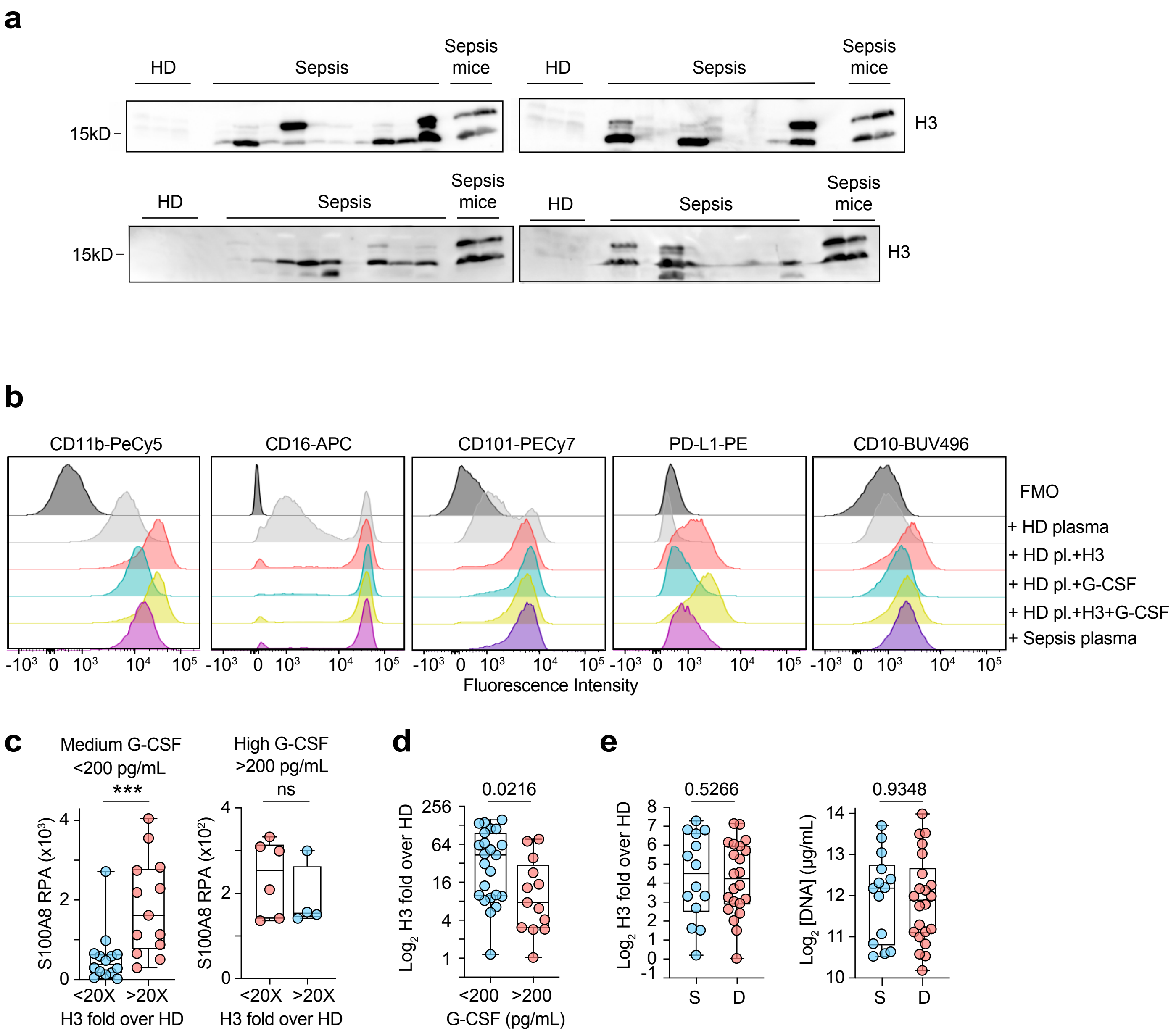

Supplementary Figure 12. Sepsis patient plasma analysis and impact on neutrophil surface marker expression. a Western immunoblotting for histone H3 in the plasma of healthy donors (HD, n=3) and sepsis patients (n=36). **b** Representative histograms of flow cytometry analysis of surface markers on healthy donor CD15$^+$CD66b$^+$ neutrophils incubated with HD or sepsis patient plasma alone or HD plasma supplemented with histone H3 or G-CSF alone or in combination for 1000 min. Panel: CD11b-PECy5, CD16-APC, CD101-PECy7, PD-L1-PE, CD10-BUV496. **c** Concentration of S100A8 in plasmas containing medium (<200 pg/mL, left panel) or high (>200 pg/mL, right panel) G-CSF levels, grouped based on medium (<20X fold over HD; n=26) or high (>20X fold over HD; n=10 donors) histone H3 plasma concentrations. **d** Histone H3 levels in the plasma of sepsis patients segregated into high and medium G-CSF containing groups. **e** Histone H3 and DNA levels in the plasma of patients that survived (S) or died (D). Statistical analysis by unpaired two-sided parametric t-test (* p<0.05, ** p<0.01, *** p<0.001, **** p<0.0001).

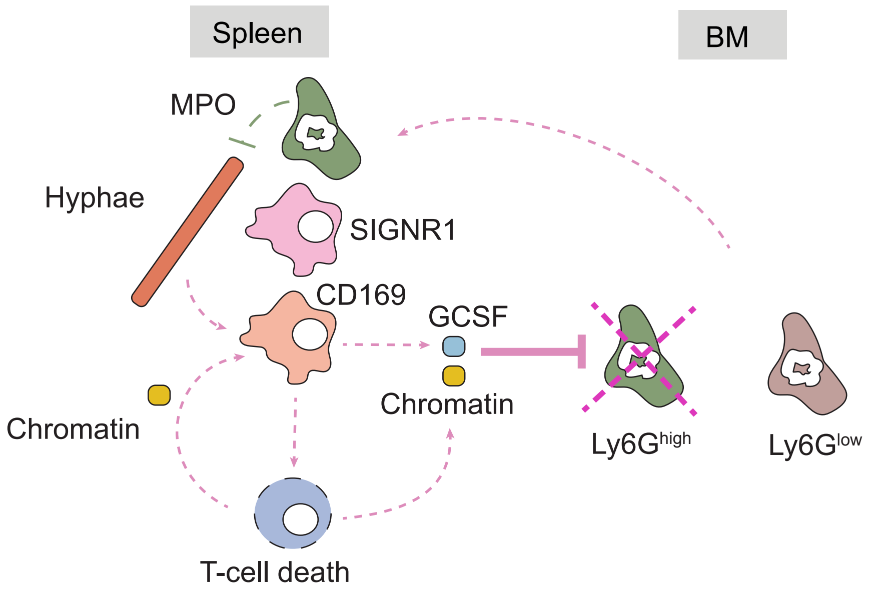

Supplementary Figure 13. SINGR1-mediated microbe capture promotes hyperinflammation and immune dysfunction. Schematic representation depicting the pathogenic feedback mechanism that enables pathogens to exploit microbial capture in the spleen, in order to degrade immune defense and trigger sepsis. Neutrophil-derived myeloperoxidase (MPO) controls microbes captured by spleen marginal zone SIGNR1^{+} macrophages through the release of MPO-derived oxidants. Microbial infiltration into the inner CD169^{+} macrophage layer, triggers T cell death and the release of extracellular chromatin in a T cell dependent manner. Synergistic activation of CD169 macrophages by histone and fungal hypahe induces G-CSF alongside other cytokines. G-CSF and histones progressively eliminate mature Ly6G^{high} neutrophils by selectively shortening their lifespan, leading to a prevalence of immature Ly6G^{low} neutrophils with defective ROS production that are unable to control hyphal growth in the spleen and other organs. This feedback loop promotes immune dysfunction in both the innate and the adaptive immune compartments, leading to hyperinflammation, immune deficiency and sepsis.

Supplementary Table 1. Antibody list

Application	Target species	Target protein	Fluorophore/label
FACS Neutrophil panel	Mouse	CD3	PerCP-Cy5.5
FACS Neutrophil panel	Mouse	CD19	PerCP-Cy5.5
FACS Neutrophil panel	Mouse	CD11b	PE-Cy7
FACS Neutrophil panel	Mouse	Ly6G	APC
FACS Neutrophil panel	Mouse	Ly6C	BV711
FACS Neutrophil panel	Mouse	G-CSFR	Unconjugated
FACS Neutrophil panel	Rat	IgG	AF488
FACS Neutrophil panel	Mouse	MPO	FITC
FACS HSC panel	Mouse	CD3	Biotin
FACS HSC panel	Mouse	CD4	Biotin
FACS HSC panel	Mouse	CD8	Biotin
FACS HSC panel	Mouse	Gr-1	Biotin
FACS HSC panel	Mouse	B220	Biotin
FACS HSC panel	Mouse	TER119	Biotin
FACS HSC panel	Mouse	c-KIT	BV711
FACS HSC panel	Mouse	Sca-1	BUV395
FACS HSC panel	Mouse	CD16/32	PE
FACS HSC panel	Mouse	CD34	FITC
FACS Neutrophil panel	Human	CD10	BUV496
FACS Neutrophil panel	Human	CD11b	PE-Cy5
FACS Neutrophil panel	Human	CD15	BV510
FACS Neutrophil panel	Human	CD16	APC
FACS Neutrophil panel	Human	CD66b	FITC
FACS Neutrophil panel	Human	CD101	PE-Cy7
FACS Neutrophil panel	Human	PD-L1	PE
IHC primary antibody	Mouse	B220	BV421
IHC primary antibody	*Candida*	recognizes various proteins in [illegible]	Unconjugated
IHC primary antibody	Mouse	CD3	AF488
IHC primary antibody	Mouse	CD4	AF488
IHC primary antibody	Mouse	CD169	AF647
IHC primary antibody	Mouse	F4/80	Unconjugated
IHC primary antibody	Mouse	Ly6G	AF647
IHC primary antibody	Mouse	MARCO	Unconjugated
IHC primary antibody	Mouse	MPO	Unconjugated
IHC primary antibody	Mouse	SIGNR1	AF488
IHC primary antibody	Mouse	SIGNR1	Biotin
IHC primary antibody	Mouse	TCR-β	FITC
IHC primary antibody	-	MDA	Unconjugated
IHC primary antibody	Mouse	cleaved-caspase3	Unconjugated
IHC primary antibody	Mouse	cleaved-caspase8	Unconjugated
IHC primary antibody	*S. aureus*	-	Unconjugated
IHC secondary antibody	Rabbit	IgG	AF488/AF568/AF647
IHC secondary antibody	Goat	IgG	AF488/AF568/AF648
IHC secondary antibody	Rat	IgG	AF488/AF568/AF649
In vivo Neu depletion	Mouse	Ly6G	Unconjugated
In vivo Neu depletion	-	IgG2a κ isotype control	Unconjugated
In vivo SIGNR1-block	Mouse	SIGNR1	Unconjugated
In vivo SIGNR1-block	-	IgG isotype control	Unconjugated
In vivo G-CSF neutralization	Mouse	G-CSF	Unconjugated

In vivo G-CSF neutralization	-	IgG2a κ isotype control	Unconjugated
In vivo histone neutralization	Multiple	Histone H3	Unconjugated
In vivo histone neutralization	Multiple	Histone H4	Unconjugated
In vivo histone neutralization	-	rabbit IgG isotype control	Unconjugated
In vitro H3 neutralization	Human	Histone H3	Unconjugated
In vitro G-CSF neutralization	Human	G-CSF	Unconjugated

Clone	Dilution	Manufacturer	Catalogue number
17A2	1:200	BioLegend	100218
6D5	1:200	BioLegend	115534
M1/70	1:500	BioLegend	101216
1A8	1:200	BioLegend	127614
HK1.4	1:500	BioLegend	128037
680206	1:50	R&D	MAB6039
Polyclonal	1:100	Invitrogen	A21208
8F4	1:50	Hycult Biotech	HM1051F-100UG
145-2C11	1:50	BioLegend	100304
RM4-5	1:50	BioLegend	100406
53-6.7	1:50	BioLegend	100704
RB6-8C5	1:50	BioLegend	108404
RA3-6B2	1:50	BioLegend	103204
TER-119	1:50	BioLegend	116204
2B8	1:100	BD horizon	563160
D7	1:100	BD Biosciences	566216
2.4G2	1:100	BD biosciences	553145
RAM34	1:100	BD Biosciences	560238
HI10a	1:100	BD Bioscience	741137
M1/70	1:200	BioLegend	101210
W6D3	1:200	BD Biosciences	563141
3G8	1:200	BD Biosciences	561248
G10F5	1:200	BioLegend	305103
BB27	1:200	BioLegend	331014
MIH3	1:100	BioLegend	374512
RA3-6B2	1:50	BioLegend	103239
Polyclonal	1:1000	Acris	BP1006
17A2	1:100	BioLegend	100210
GK1.5	1:100	BioLegend	100423
3D6.112	1:100	BioLegend	142408
A3-1	1:100	Abcam	ab6640
1A8	1:50	BioLegend	127610
ED31	1:50	BMA Biomedicals	T-2026
Polyclonal	1:40	R&D Systems	AF3667
eBio22D1	1:100	eBioscience	53-2093-82
ER-TR9	1:50	Abcam	ab51819
H57-597	1:100	Invitrogen	11-5961-82
Polyclonal	1:500	Abcam	ab6463
-	1:400	Cell Signaling	9661
-	1:800	Cell Signaling	8592
Polyclonal	1:800	ThermoFisher Scientific	PA1-7246
Polyclonal	1:200	Invitrogen	A21206/A10042/A31573
Polyclonal	1:200	Invitrogen	A11055/A11057/A21447
Polyclonal	1:200	Invitrogen	A21208/A11077/A21247
1A8	-	BioXCell	BE0075-1
2A3	-	BioXCell	BE0089
22D1	-	BioXCel	BE0220
Polyclonal	-	BioXCell	BE0091
9B4CSF	-	Invitrogen eBioscience	16-7353-85

2A3	-	BioXCell	BE0089
Polyclonal	-	Merck Millipore	07-690
62-141-13	-	Merck Millipore	04-858
Polyclonal	-	BioXCell	BE0095
Polyclonal	-	Millipore	06-755
EPR3203(N)(B)	-	Abcam	ab181053
