# Supplementary material for: Microbe capture by splenic macrophages triggers sepsis via T cell-death-dependent neutrophil lifespan shortening
Source: Nat Commun. 2022 Aug 9;13:4658. doi: 10.1038/s41467-022-32320-1 (PMC9361272; doi:10.1038/s41467-022-32320-1)
Supplement: Supplementary file 2 — Reporting Summary [file 41467_2022_32320_MOESM2_ESM.pdf]

Double-blind peer review submisions: write DBPR and your manuscript number here

nature portfolio

Corresponding author(s): Venizelos Papayannopoulos

Last updated by author(s): 2022-06-07

Reporting Summary

Nature Portfolio wishes to improve the reproducibility of the work that we publish. This form provides structure for consistency and transparency in reporting. For further information on Nature Portfolio policies, see our Editorial Policies and the Editorial Policy Checklist.

Statistics

For all statistical analyses, confirm that the following items are present in the figure legend, table legend, main text, or Methods section.

n/a	Confirmed	
☐	☒	The exact sample size (*n*) for each experimental group/condition, given as a discrete number and unit of measurement
☐	☒	A statement on whether measurements were taken from distinct samples or whether the same sample was measured repeatedly
☐	☒	The statistical test(s) used AND whether they are one- or two-sided<br>*Only common tests should be described solely by name; describe more complex techniques in the Methods section.*
☒	☐	A description of all covariates tested
☐	☒	A description of any assumptions or corrections, such as tests of normality and adjustment for multiple comparisons
☐	☒	A full description of the statistical parameters including central tendency (e.g. means) or other basic estimates (e.g. regression coefficient) AND variation (e.g. standard deviation) or associated estimates of uncertainty (e.g. confidence intervals)
☐	☒	For null hypothesis testing, the test statistic (e.g. *F*, *t*, *r*) with confidence intervals, effect sizes, degrees of freedom and *P* value noted<br>*Give P values as exact values whenever suitable.*
☒	☐	For Bayesian analysis, information on the choice of priors and Markov chain Monte Carlo settings
☒	☐	For hierarchical and complex designs, identification of the appropriate level for tests and full reporting of outcomes
☒	☐	Estimates of effect sizes (e.g. Cohen's *d*, Pearson's *r*), indicating how they were calculated

Our web collection on statistics for biologists contains articles on many of the points above.

Software and code

Policy information about availability of computer code

Data collection	n/a
Data analysis	n/a

For manuscripts utilizing custom algorithms or software that are central to the research but not yet described in published literature, software must be made available to editors and reviewers. We strongly encourage code deposition in a community repository (e.g. GitHub). See the Nature Portfolio guidelines for submitting code & software for further information.

Data

Policy information about availability of data

All manuscripts must include a data availability statement. This statement should provide the following information, where applicable:

- Accession codes, unique identifiers, or web links for publicly available datasets
- A description of any restrictions on data availability
- For clinical datasets or third party data, please ensure that the statement adheres to our policy

The high throughput RNA sequencing expression profile data is publically made available and can be accessed at NCBI Gene Expression Omnibus (GSE160301) and the token (ydyjocqyjpczjgt). The mass spectrometry proteomics data have been deposited to the ProteomeXchange Consortium via the PRIDE partner repository with the dataset identifier PXD034391. All other source data are provided with this paper.

Field-specific reporting

Please select the one below that is the best fit for your research. If you are not sure, read the appropriate sections before making your selection.

- [x] Life sciences
- [ ] Behavioural & social sciences
- [ ] Ecological, evolutionary & environmental sciences

For a reference copy of the document with all sections, see nature.com/documents/nr-reporting-summary-flat.pdf

Life sciences study design

All studies must disclose on these points even when the disclosure is negative.

Sample size	Sample sizes were determined based on pilot studies, published comparative studies and expert experience.
Data exclusions	No data was excluded in this study.
Replication	Most experiments were validated with repeat experiments. Few exceptions include costly experiments, such as Cytokine arrays, proteomics and RNAsequencing were biological replicates were employed. Sufficient sample size was included and when possible, samples were processed simultaneously to prevent batch variations
Randomization	Treatment groups in animal experiments were mixed within the same cage, to prevent variation among different cages, and groups were assigned randomly. Proteomic analysis was also randomized to prevent run bias.
Blinding	N/A

Reporting for specific materials, systems and methods

We require information from authors about some types of materials, experimental systems and methods used in many studies. Here, indicate whether each material, system or method listed is relevant to your study. If you are not sure if a list item applies to your research, read the appropriate section before selecting a response.

Materials & experimental systems

n/a	Involved in the study	
☐	☒	Antibodies
☒	☐	Eukaryotic cell lines
☒	☐	Palaeontology and archaeology
☐	☒	Animals and other organisms
☐	☒	Human research participants
☐	☒	Clinical data
☒	☐	Dual use research of concern

Methods

n/a	Involved in the study	
☒	☐	ChIP-seq
☐	☒	Flow cytometry
☒	☐	MRI-based neuroimaging

Antibodies

Antibodies used

Neutrophil depletion: anti-Ly6G (clone 1A8; BioXCell; cat. BE0075-1) or rat IgG2a κ isotype control (clone 2A3; BioXCell; cat. BE0089).
SIGNR1 blocking: anti-SIGNR1 (clone 22D1; BioXCel; cat. BE0220) or polyclonal Armenian hamster IgG isotype control (BioXCell; cat.. BE0091).
G-CSF neutralization: anti-G-CSF (clone 9B4CSF; Invitrogen eBioscienceTM; cat. 16-7353-85) or rat IgG2a κ isotype control (clone 2A3; BioXCell; cat. BE0089).
Histone neutralisation experiments: anti-Histone H3 (Merck Millipore; cat. 07-690) and anti-Histone H4 antibodies (clone 62-141-13; Merck Millipore; cat. 04-858) or control polyclonal rabbit IgG (BioXCell; cat. BE0095).
In vitro blocking: anti-human H3 (polyclonal; Millipore; cat. 06-755) and anti-human G-CSF (clone EPR3203(N)(B); Abcam; Cat. ab181053).
Immunohistochemistry primary antibodies: anti-B220 (clone RA3-6B2; BioLegend; dilution 1/50), anti-Candida (polyclonal; Acris; cat. BP1006; dilution 1/1000), anti-CD3 (clone 17A2; BioLegend; dilution 1/100), anti-CD4 (clone GK1.5; BioLegend; dilution 1/100), anti-CD169 (clone 3D6.112; BioLegend; dilution 1/100), anti-F4/80 (clone BM8; BioLegend; 1/100), anti-Ly6G (clone 1A8; BioLegend; dilution 1/50), anti-MARCO (clone ED31; BMA Biomedicals; dilution 1/50), anti-MPO (polyclonal; R&D Systems; cat. AF3667; dilution 1/40), anti-SIGNR1 (clone eBio22D1; eBioscience; dilution 1/100), anti-TCR-β (clone H57-597; eBioscience; dilution 1/100), anti-cleaved-caspase 3 (Cell Signaling; cat. 9661; dilution 1/400) and anti-cleaved-caspase 8 (Cell Signaling; cat. 8592; dilution 1/800).
Immunohistochemistry secondary antibodies: donkey anti-rabbit IgG (polyclonal; Invitrogen; dilution 1/200), donkey anti-goat IgG (polyclonal; Invitrogen; dilution 1/200) and goat anti-rat IgG (polyclonal; Invitrogen; dilution 1/200).
Flow cytometry mouse neutrophil panel: anti-CD3 (clone 17A2; BioLegend; dilution 1/200), anti-CD19 (clone 6D5; BioLegend; dilution 1/200), anti-CD11b (clone M1/70; BioLegend; dilution 1/500), anti-Ly6G (clone 1A8; BioLegend; dilution 1/200), anti-Ly6C (clone HK1.4; BioLegend; dilution 1/500), anti-G-CSFR primary (clone 680206; R&D; dilution 1/50) and anti-rat secondary (polyclonal; Invitrogen; dilution 1/100).

Flow cytometry mouse HSC panel: CD3 (clone 145-2C11; BioLegend; dilution 1/50), CD4 (clone RM4-5; BioLegend; dilution 1/50), CD8 (clone 53-6.7; BioLegend; dilution 1/50), Gr-1 (clone RB6-8C5; BioLegend; dilution 1/50), B220 (clone RA3-6B2; BioLegend; dilution 1/50), TER119 (clone TER-119; BioLegend; dilution 1/50), c-KIT (clone 2B8; BD horizon; dilution 1/100), Sca-1 (clone D7; BD Biosciences; dilution 1/100), CD16/32 (clone 2.4G2; BD biosciences; 1/100), CD34 (clone RAM34; BD Biosciences; dilution 1/100).
Flow cytometry human neutrophil panel: anti-CD10 (clone HI10a; BD Bioscience; 1/100), anti-CD11b (clone M1/70; BioLegend; dilution 1/200), anti-CD15 (clone W6D3; BD Biosciences; 1/200), anti-CD16 (clone 3G8; BD Biosciences; 1/200), anti-CD66b (clone G10F5; BioLegend; dilution 1/200), anti-CD101 (clone BB27; BioLegend; dilution 1/200), anti-PD-L1 (clone MIH3;BioLegend; dilution 1/100) and MPO (clone 8F4; Hycult Biotech; dilution 1/50).

Validation

Clones were selected based on experience or published literature, and validated before use for experimental data.

Animals and other organisms

Policy information about studies involving animals; ARRIVE guidelines recommended for reporting animal research

Laboratory animals	All experiments were conducted with age-matched and cage-controlled, 8 to 12-week-old female wild-type (WT) C57BL/6J, MPO-/- (Mpotm1Lus), Cybb-/- (Cybbtm1Din), TCRα-/- (Tcratm1Phi), Rag2-/- (Rag2tm1Fwa), G-CSF-/-(Csf3tm1Ard) and FVB/NJ Mus musculus mice.
Wild animals	This study did not involve wild aninals
Field-collected samples	This study did not involve samples collected in the field.
Ethics oversight	Peripheral blood was isolated from consenting healthy adult volunteers, according to approved protocols of the ethics board of the Francis Crick Institute and the Human Tissue act. Sepsis patient samples were provided by the Hannover Medical School approved the ethics committee under the study protocol (No. 2786-2015). Written informed consent was obtained from participants or authorized representatives. The study was performed in accordance with the ethical standards laid down in the 1964 Declaration of Helsinki and its later amendments.

Note that full information on the approval of the study protocol must also be provided in the manuscript.

Human research participants

Policy information about studies involving human research participants

Population characteristics	Healthy adult volunteers donated peripheral blood for in vitro experiments, and septic patients were included for plasma analysis
Recruitment	All donors were recruited voluntarily and consented to the study objectives.
Ethics oversight	Ethics board of the Francis Crick Institute, the Human Tissue act and the Hannover Medical School ethics committee.

Note that full information on the approval of the study protocol must also be provided in the manuscript.

Clinical data

Policy information about clinical studies
All manuscripts should comply with the ICMJEguidelines for publication of clinical research and a completedCONSORT checklist must be included with all submissions.

Clinical trial registration	NCT04231994
Study protocol	No. 2786-2015
Data collection	*Describe the settings and locales of data collection, noting the time periods of recruitment and data collection.*
Outcomes	*Describe how you pre-defined primary and secondary outcome measures and how you assessed these measures.*

Flow Cytometry

Plots

Confirm that:

- [x] The axis labels state the marker and fluorochrome used (e.g. CD4-FITC).
- [x] The axis scales are clearly visible. Include numbers along axes only for bottom left plot of group (a 'group' is an analysis of identical markers).
- [x] All plots are contour plots with outliers or pseudocolor plots.
- [x] A numerical value for number of cells or percentage (with statistics) is provided.

Methodology

Sample preparation

Peripheral venous blood was collected into S-Monovette 7.5 ml sodium heparin tubes (Laborimpex; cat. 01.1613.100) and kept at room temperature (RT) until start of neutrophil isolation. 5ml whole blood was carefully layered on top of 5ml prewarmed Histopaque 1119 (Sigma-Aldrich; cat. 11191-100ml) at 37oC in a 15ml Falcon tube. Tubes were then centrifuged for 20 min at 800xg at RT, with acceleration speed 7 and deceleration speed 3. The transparent top layer containing plasma was placed in a new Falcon tube and spun for 10 min at 300xg at RT to ensure acellularity. The subsequent layers containing peripheral blood mononuclear cells (PBMCs) and neutrophils were collected separately in new 15ml Falcon tubes and washed in Cytiva HyClone Hank's balanced salt solutions (HBSS) without calcium, magnesium or henol red (Fisher Scientific; cat. SH3058801) supplemented with 10mM HEPES (Invitrogen; H0887-100ml) 0.1% plasma and spun for 10min at 300xg at RT. PBMCs and neutrophils were resuspended in 2ml washing buffer before being further fractionated on a discontinuous Percoll (GE Healthcare; cat. 17-5445-02) gradient. Neutrophils were overlayed on a density gradient in a 15ml Falcon tube containing 2ml of each of the following densities: 1105 g/ml (85%), 1100 g/ml (80%), 1093 g/ml (75%), 1087 g/ml (70%), and 1081 g/ml (65%), containing the highest density at the bottom of the tube. Neutrophils were then centrifuged for 20 min at 800xg, with acceleration speed 7 and deceleration speed 3. Neutrophil enriched layers (75-65% layer) were collected and washed in washing buffer, and spun for 10min at 300xg at RT. Neutrophils were resuspended in 1ml washing buffer and manually counted with a cell-counting chamber.

For murine blood collection, mice were euthanised with pentobarbital (600mg/kg) with mepivacaine hydrochloride (20mg/ml). When animals became irresponsive, blood was collected via cardiac puncture using a 25Gx5/8" needle (BD; cat. 300600) and an 1ml syringe (BD; 303172). After collection, the needle was disposed and blood sample put in a blood collection 1.3ml microvette tube with a lithium-heparin ring (Sarstedt; cat. 41/09). The heparin tube was gently inverted 10 times and left at RT until proceeding. To separate the leukocytes from plasma and remove a large proportion of red blood cells, a maximum of 300µl whole blood was overlayed on 1ml Histopaque-1119 (Sigma; cat. 11191-100ML) at RT in an Eppendorf and spun for 20min at 800xg RT, with acceleration speed 7 and deceleration speed 3. The top transparent plasma layer was carefully collected without collecting cells. The remaining supernatant containing the leukocytes and was put in a 15ml Falcon tube, topped up with PBS 3% FCS and spun for 10min at 300xg at 4oC. Pellet was resuspended in 1ml PBS 3% FCS and left on ice until proceeding.

For leukocytes from murine spleens, kidney and bone marrow (tibia and femur), the organs were collected organs were kept in 1ml sterile PBS 3% FCS on ice. For splenocytes, spleens were gently meshed in PBS 3% FCS (Sigma) using a 40µM nylon mesh cell strainer (Fisher Scientific; cat. 22363547) and a plunger from a 2ml syringe (BD; cat. 307727), to prepare single cell suspensions. Cell suspension was collected in 15ml Falcon tubes, topped up with PBS 3% FCS and left on ice until proceeding. For kidneys cells, organs were chopped up with a scalpel and incubated in 10ml digestion medium containing 0.2mg/ml Liberase TL (Roche Diagnostics; cat. 05401020001) and 0.1mg/ml DNase I (Roche; cat. 11284932001) in a 50ml Falcon for 20 minutes while shaking at 37oC. The digestion medium and tissues were filtered and meshed through an 100µM nylon mesh cell strainer (Fisher Scientific; cat. 352360) and a plunger from a 2ml syringe, to prepare single cell suspensions in a new 50ml Falcon tube. Cell suspension was spun for 10 min at 300xg at RT and the pellet was resuspended in 50ml PBS 3% FCS to wash away the digestion buffer. The samples were spun again for 10 min at 300xg at RT and the pellet was resuspended in 8ml or 40% Percoll (GE Healthcare; cat. 17-5445-02), which was carefully overlayed on top of 3ml 70% Percoll in a 15ml Falcon tube. This step was performed to eliminate debris and non-leukocytes. The Percoll gradient was spun for 20min at 800xg RT, with acceleration speed 7 and deceleration speed 3. The top layer represents fat and debris, which is carefully collected and discarded without interrupting inferior layers. Leukocytes were present mostly in the interphase between 40% and 70%, which were then collected and put in a 15ml Falcon tube, topped up with PBS 3% FCS and spun down for 10min at 300xg at 4oC. The cells were then washed and spun twice more in PBS 3% FCS and kept on ice until proceeding.

For bone marrow cells femur and tibia were cut with surgical scissors on one end of the bone and placed in a perforated 0.5ml Eppendorf inside a 1.5ml Eppendorf tube. Eppendorf's were spun for 15 seconds at 10.000xg at RT. The pellet was resuspended in PBS 3% FCS and kept on ice until proceeding. To deplete erythrocytes, all cell suspensions were spun for 10min at 300xg in 4oC and the pellet was resuspended in 2ml ammonium-chloride-potassium (ACK; Gibco; cat. A10492-01) lysing buffer and incubated for 3min at RT. The tubes were topped up with PBS 3% FCS and kept on ice until proceeding. All samples were then filtered again with an 40µM nylon mesh cell strainer (Fisher Scientific; cat. 22363547) and were then spun for 10min at 300xg at 4oC. The pellets were resuspended in 300-1000µl PBS 3% FCS and counted.

For flow cytometry, single cell suspensions were obtained as indicated previously. Maximum 5x106 cells were plated in a U-bottom 96-well plate and spun for 10min at 300xg at 4oC. Cells were washed by resuspending the pellets in 200µl PBS and centrifuging for 10min at 300xg at 4oC. Cells were then stained for 25 mins at 4oC in the dark in 100µl PBS with fixable live-dead stain and fluorescently-labelled antibodies. After staining, 100µl PBS 3% FCS was added to each well and the plate was spun for 10min at 300xg at RT. Cells were then washed once more in 200µl PBS 3% FCS and spun. The pellet was resuspended in 100µl 4% PFA (Thermo Scientific; cat. 28908) in PBS for fixation 25min at 4oC in the dark. Then, 100µl PBS 3% FCS was added to each well and the plate was spun for 10min at 300xg at RT. Cells were then resuspended in 200ml PBS, stored at 4oC and analysed within 2 days. Cells were filtered with 40µM cell strainers or 50µM filter mesh before analysis.

When intracellular staining was required, Foxp3 / transcription factor staining buffer set (ThermoFisher Scientific eBiosciences; cat. 00-5523-00) was used. Cells were fixed in fix-perm buffer instead of 4% PFA. Washed fixed cells were then first left in 50µl 1x permeabilization (perm.) buffer for 10minutes at 4oC in the dark. Then 50ul of 2x MPO (clone 8F4; Hycult Biotech; dilution 1/50) in 1x permeabilization buffer was added and incubated for 25min at 4oC in the dark. After secondary staining, 100µl perm. buffer was added to each well and the plate was spun for 10min at 300xg at RT. Cells were then washed twice in 200µl perm buffer and spun. Cells were then resuspended in 200ml PBS, stored at 4oC and analysed within 2 days.

For the Vβ T cell analysis, single cell suspensions were obtained as indicated above and the mouse Vβ TCR Screening Panel

	(BD Pharmingen; cat. 557004) was used for staining. 15 stainings, one for each Vβ TCR, wereperformed for each sample and the staining protocol was identical as indicated above.<br><br>For sorting, single cell suspensions from spleen and bone marrow were obtained as described prior and enriched with the EasySep mouse neutrophil enrichment kit (Stemcell Technologies; cat. 19762), following the manufacturer's instructions. Enriched cell-suspensions were stained in 2ml (spleen) or 0.5ml (BM) PBS 2% FCS with life/dead staining DAPI (Invitrogen; cat. D1306; 1:1000) and the fluorescently labelled antibodies indicated above for 25min at 4oC in the dark, in a 15ml Falcon tube. After staining, the tube was topped up with PBS 2% FCS and spun for 10min at 300xg at 4oC. The pellet was resuspended in 1-2ml PBS 2% FCS, to obtain an approximate cell concentration of 10x106 cells/ml. Cells were filtered before sorting, and were sorted with either a 70µm nozzle (for RNAseq and Giemsa stainings) or a 100µm nozzle (for in vitro culture experiments). Sorted cells were collected in sterile FACS tubes or 15ml Falcons containing 0.5-1ml 100% sterile FCS. All sortings were performed on a BD FACS Aria cell sorter.
Instrument	BD LSR Fortessa
Software	FlowJo software v10
Cell population abundance	For flow cytometry, 150-300ul murine blood, 5 milion splenocytes/kidney cells or 10 milion bone marrow cells were stained. For sorting of splenic Ly6Glow/high cells for RNAsequencing, whole spleens (50-100M) were enriched for neutrophils with an EasySep isolation kit, and 10K-1M cells were eventually sorted. Purity of cells was evaluated after and was 95-100%.
Gating strategy	For mouse neutrophil staining: singlet live CD3-CD19-CD11b+Ly6Ghigh/Ly6Glow<br>For mouse HSC staining: singlet live lineage-(CD3-CD4-CD8-GR1-B220-TER119-)c-KIT+Sca-1- and CD16/32low/- (MEPs), CD16/32intermediateCD34+ (CMPs) or CD16/32high (GMPs).<br><br>To determine marker signal positivity for important populations, FMO's were included, such as GCSFR, HSC dump gate, CD16/32 and CD34.

☒ Tick this box to confirm that a figure exemplifying the gating strategy is provided in the Supplementary Information.
